# Serial block-face scanning electron microscopy reveals neuronal-epithelial cell fusion in the mouse cornea

**DOI:** 10.1371/journal.pone.0224434

**Published:** 2019-11-13

**Authors:** Justin A. Courson, Ian Smith, Thao Do, Paul T. Landry, Aubrey Hargrave, Ali R. Behzad, Sam D. Hanlon, Rolando E. Rumbaut, C. Wayne Smith, Alan R. Burns

**Affiliations:** 1 University of Houston, College of Optometry, Houston, TX, United States of America; 2 King Abdullah University of Science and Technology (KAUST), Core Labs, Thuwal, Saudi Arabia; 3 Baylor College of Medicine, Children’s Nutrition Center, Houston, TX, United States of America; 4 Center for Translational Research on Inflammatory Diseases (CTRID), Michael E. DeBakey Veterans Affairs Medical Center, Houston, TX, United States of America; University of Florida, UNITED STATES

## Abstract

The cornea is the most highly innervated tissue in the body. It is generally accepted that corneal stromal nerves penetrate the epithelial basal lamina giving rise to intra-epithelial nerves. During the course of a study wherein we imaged corneal nerves in mice, we observed a novel neuronal-epithelial cell interaction whereby nerves approaching the epithelium in the cornea fused with basal epithelial cells, such that their plasma membranes were continuous and the neuronal axoplasm freely abutted the epithelial cytoplasm. In this study we sought to determine the frequency, distribution, and morphological profile of neuronal-epithelial cell fusion events within the cornea. Serial electron microscopy images were obtained from the anterior stroma in the paralimbus and central cornea of 8–10 week old C57BL/6J mice. We found evidence of a novel alternative behavior involving a neuronal-epithelial interaction whereby 42.8% of central corneal nerve bundles approaching the epithelium contain axons that fuse with basal epithelial cells. The average surface-to-volume ratio of a penetrating nerve was 3.32, while the average fusing nerve was smaller at 1.39 (p ≤ 0.0001). Despite this, both neuronal-epithelial cell interactions involve similarly sized discontinuities in the basal lamina. In order to verify the plasma membrane continuity between fused neurons and epithelial cells we used the lipophilic membrane tracer DiI. The majority of corneal nerves were labeled with DiI after application to the trigeminal ganglion and, consistent with our ultrastructural observations, fusion sites recognized as DiI-labeled basal epithelial cells were located at points of stromal nerve termination. These studies provide evidence that neuronal-epithelial cell fusion is a cell-cell interaction that occurs primarily in the central cornea, and fusing nerve bundles are morphologically distinct from penetrating nerve bundles. This is, to our knowledge, the first description of neuronal-epithelial cell fusion in the literature adding a new level of complexity to the current understanding of corneal innervation.

## Introduction

The cornea is the most highly innervated tissue in the mammalian body [1]. The nerves of the cornea provide autonomic responses such as tearing and blinking and assist in maintaining corneal epithelial homeostasis through the release of trophic factors [2]. Sympathetic innervation comes from nerve fibers originating in the superior cervical ganglion while sensory information is transmitted from the corneal epithelium to cell bodies located in the trigeminal ganglion, [3–6]. It is well-established that corneal stromal nerves enter the cornea in the peripheral stroma and travel horizontally before branching to give rise to vertical axons that penetrate the epithelial basal lamina [7, 8]. Penetrated nerves ramify shortly after entering the corneal epithelium (in a process known as leash formation), and these ramifications constitute the sub-basal plexus. Axons in the sub-basal plexus travel anteriorly and laterally between the wing and superficial-squamous cells of the corneal epithelium, after which they give rise to the epithelial nerve plexus in addition to axon terminals [9, 10]. Corneal innervation is a dynamic process, constantly changing as a result of aging and in response to pathology or injury [11]. The mechanisms by which corneal nerve patterning is regulated are not well established.

In addition to data gathered from studies on neurotransmission, our understanding of corneal innervation is largely based on light and electron microscopic imaging. While transmission electron microscopy (TEM) makes it possible to appreciate corneal nerve ultrastructure from single ultrathin sections, it provides only a two-dimensional perspective [12]. For a three-dimensional context, serial sections are needed and while serial sectioning using TEM is possible, the technical challenge of collecting serial sections is demanding and typically limits three-dimensional (3D) reconstructions to less than 50 serial images spanning a depth of no more than 5 microns [13]. To our knowledge, no serial sectioning electron microscopy studies have been reported on the nerves of the cornea.

With the advent of a relatively new technique known as serial block-face scanning electron microscopy (SBF-SEM) it is now possible to collect 3D ultrastructural data with relative ease. Routine automated collection of a thousand or more serially-registered images spanning a depth of 50 to 100 microns allows for superior 3D reconstructions and improved ultrastructural interpretation [14]. In addition to providing the ability to produce 3D reconstruction of tissue at an ultrastructural level, the context provided by serial section imaging allows for the identification of complex cell-cell interactions at an ultrastructural level that cannot be seen using light microscopy or single section electron microscopy. As a result, SBF-SEM has been applied across a great deal of tissue in the literature, but has yet to be used to study corneal nerves.

The purpose of the current study was to use the 3D capabilities of SBF-SEM to directly examine stromal nerve penetrations into the corneal epithelium of mice. Shortly after initiating the study, we observed for the first time a novel neuronal-epithelial cell interaction in which stromal nerves approach the epithelium and fuse with basal epithelial cells. Herein we use SBF-SEM to describe and compare two types of neuronal-epithelial interactions, simple neuronal penetration into the corneal epithelium and the novel fusion event that also occurs between stromal axons and basal epithelial cells.

## Materials and methods

### Animals

Male C57BL/6J mice aged 8–10 weeks were purchased from Jackson Labs (Sacramento, CA) and housed at the University of Houston, College of Optometry (UHCO). All animals were handled according to the guidelines described in the Association for Research in Vision and Ophthalmology Statement for the Use of Animals in Vision and Ophthalmic Research and the University of Houston College of Optometry animal handling guidelines. All animal procedures were approved by the University of Houston Animal Care and Use Committee (IACUC number: 16–005).

### Electron microscopy

#### Tissue processing

Mice were euthanized by CO_2_ asphyxiation followed by cervical dislocation. Tissue fixation and resin-embedding were performed as previously described [15, 16]. Briefly, following enucleation, the eyes were placed in primary fixative (0.1M sodium cacodylate buffer containing 2.5% glutaraldehyde and 20mM calcium chloride) for 2 hours at room temperate. Fixed corneas, with the limbus intact, were carefully excised from the whole eye and cut into four equal quadrants. These quadrants were then washed in buffer before serial contrasting in potassium ferrocyanide, osmium tetroxide, thiocarbohydrazide and osmium tetroxide. The contrasted tissue was stained in uranyl acetate at 4⁰C overnight before being placed in a lead aspartate solution for 30 minutes at 60⁰C. Finally, the tissue was dehydrated through an acetone series before embedding in Embed 812 resin (Embed-812, Electron Microscopy Sciences, Hatsfield, PA) containing Ketjenblack (Ketjenblack EC600JD, Lion Specialty Chemicals Co., Tokyo) in order to reduce tissue charging [17]. The block-face was trimmed to a 1 mm x 1 mm size and the tissue block was then glued to an aluminum specimen pin, and covered in silver paint to further reduce charging.

#### Serial Block-Face Scanning Electron Microscopy (SBF-SEM)

Tissue blocks were sectioned using a Gatan 3View2 system (Gatan, Pleasanton, CA) mounted in a Mira 3 field emission scanning electron microscope (SEM, Tescan, Pittsburgh, PA). Back scatter electron (BSE) detection was used to image the block-face. Serial imaging was conducted under high vacuum (0.047 Pa) using a Schottky emitter and an accelerating voltage of 8–21 keV. Imaging under high vacuum has the effect of decreasing noise in collected images, but introduces the potential for tissue charging. However, the inclusion of Ketjenblack to the resin greatly diminishes the capacity for charging within the tissue. This allowed us to image our tissue under conditions that normally result in unacceptably charged images. Beam intensity ranged from 5–7 on a scale ranging from 1–20, with a pixel dwell time of 32 μs, and a spot size of 4–7 nm. Resolution improves with smaller spot sizes [18]. With a spot size of 4–7 nm the plasma membrane of cells and organelles is clearly visible as a single electron dense structure (S1 Fig). The z-step distance between each serial image in these stacks was 100 nm. Magnification ranged from 3000-5500x and pixel size from 4–15 nm.

The central cornea was defined as having a diameter of 2 mm; the peripheral cornea occupied the region (1.5 mm) between the central cornea and the limbal vasculature. The block-face was monitored at low magnification for stromal nerves that approached the corneal epithelium at which point high magnification was used to document nerve-epithelial interactions (i.e., penetration and fusion). Image stacks were post-processed for spatial drift removal using Gatan Digital Micrograph software.

Subsequent three-dimensional segmentation and reconstruction was conducted using Amira 6.0.1 software (FEI Company, Hillsboro, OR). The contours of structures of interest were manually traced for each image in the image stack using a digitizing pen connected to a Wacom tablet. Traced profiles were used to produce three dimensional volumetric reconstructions. Volumetric data was extrapolated from these digital reconstructions using the “material statistics module” in the Amira 6.0.1 software package, and surface meshes applied via the “generate surface module” in order to create digital models of each reconstruction. Images and videos of reconstructions were generated using the “animation” module. Segmentation and reconstruction using the Amira 6.0.1 software was conducted by a four-person reconstruction team. Care was taken to reconstruct the electron translucent axons separately from the electron dense axons within each nerve bundle, this was accomplished by assigning a different material (i.e. color) to each structure of interest. The basal lamina was identified by its characteristic electron density (*lamina densa*) on the stromal face of basal epithelial cells, and neuronal mitochondria by their electron dense double membrane and size.

#### Morphometric analysis

Morphometric analysis using standard stereological techniques was performed as previously described [19, 20]. Stereology is an aspect of morphometry that takes advantage of the inherent mathematical relationships between three-dimensional objects and their two-dimensional representations (e.g., electron micrographs) [21]. These relationships are based on the reasoning of geometric probability and statistics, and the practice of using stereological grids has been used extensively over the past 50 years to obtain unbiased and accurate estimates of geometric features such as cell/organelle number, length, surface area, and volume [22–28].

**Fig 1:**
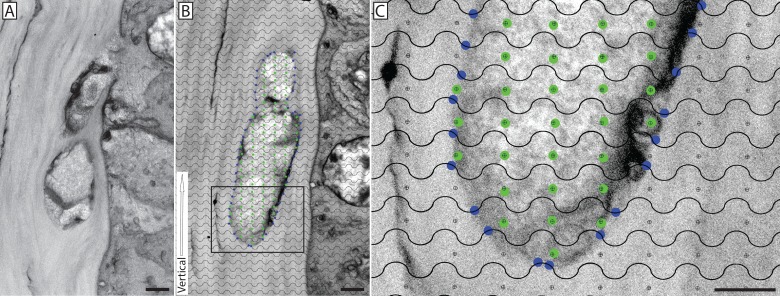
Morphometric analysis of corneal nerve surface-to-volume ratio using a cycloid grid. A single image from an SBF-SEM series showing a nerve that has fused with a basal epithelial cell (**A**). A micrograph from this series was selected at random and a cycloid grid was randomly cast onto the image while maintaining the orientation of the grid (defined by the vertical white arrow) parallel to the epithelial basal lamina (**B**). The intersection of the grid lines with the surface of the nerve bundle are marked with blue dots (surface area) while grid points falling within the nerve bundle are marked with green dots (volume); the inset, enlarged in panel (**C**), offers a magnified view of the grid. Scale bars = 2 μm.

In order to estimate the surface-to-volume ratio of fusing and penetrating nerves, a cycloid grid was used. Briefly, serial electron images were obtained of both fusing and penetrating nerve events as they approach/interact with the corneal basal epithelium (10 animals per group, with 20 nerves assessed in the fusing group and 23 nerves assessed in the penetrating group). The serial images in which the nerve is visible were identified, and a section was selected at random for analysis. Digital micrographs were analyzed in Adobe Photoshop (Adobe Systems Inc., San Jose, CA) using a cycloid grid [29]. The vertical axis of the grid was oriented in parallel to the basal lamina within each image in order to account for the anisotropic properties of the cornea. Line intersections with the nerve bundle of interest were counted, as well as target points located within the nerve bundle (**Fig 1**). In order to avoid counting line intercepts and target points within nerves located on the epithelial side of the basal lamina, a restriction line was drawn from one end of the basal lamina pore to the other and counts were only made on the stromal side of each nerve. The ratio between line intersections with the nerve and target points within the nerve was used to calculate the cell surface density, or surface-to-volume ratio using an established stereology formula:

$${Ŝ}_{V}=\frac{2∙\sum_{i=1}^{n}I_{i}}{l/p\cdot \sum_{i=1}^{n}P_{i}}$$

where *I* is the number of intersections between the grid lines and nerve bundle, *P* is the number of grid points falling within the target nerve, and *l/p* is the length of test line per grid point (corrected for magnification) [29].

Interactions between nerve and epithelium (fusion or penetration) include a discontinuity in the basal lamina. The maximum dimension of each discontinuity (i.e., basal lamina pore diameter) was identified within each image stack and measured using Fiji (ImageJ) [30].

#### Transmission Electron Microscopy (TEM)

Tissue blocks containing verified neuronal-epithelial cell fusion and nerve penetration into the basal epithelium were removed from the Gatan 3View2 system and ultra-thin sections 100 nm thick were cut, set on single slot copper grids, and imaged on a Tecnai G2 Spirit BioTWIN electron microscope (FEI Company, Hillsboro, OR). Nerve bundles were imaged and assessed for the presence of microtubules and cellular organelles.

### DiI labeling of trigeminal ganglia

#### Tissue processing

**Fig 2:**
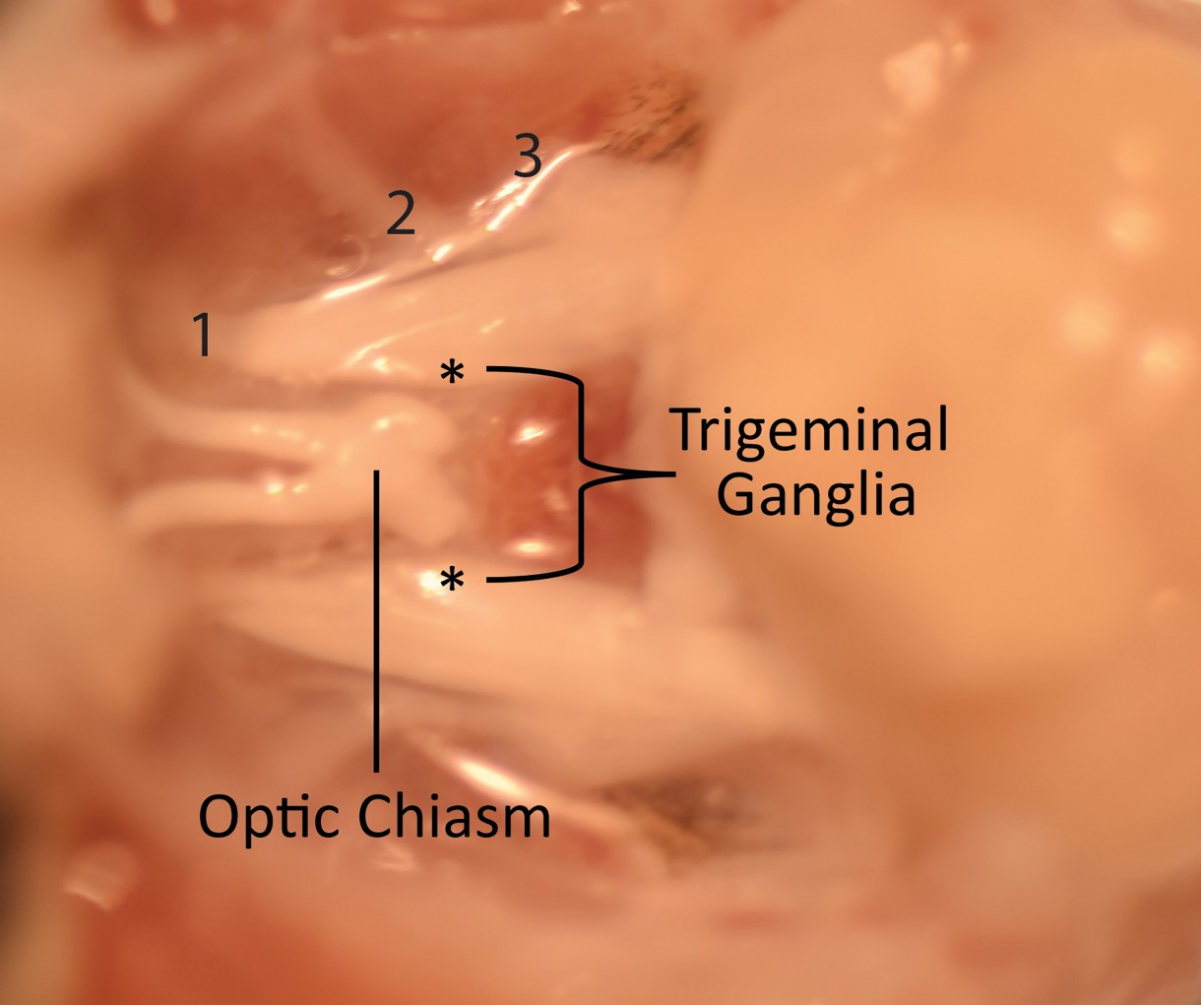
Application of DiI to the trigeminal ganglia of the mouse. A dissected view of the mouse cranial cavity showing trigeminal ganglia (*) straddling the optic chiasm. The trigeminal branches are labeled as 1, 2 and 3 and correspond to the ophthalmic, maxillary, and mandibular branches. DiI crystal was applied to the severed ophthalmic branch.

DiI crystals were placed on the trigeminal ganglia of 6 C57BL/6J mice. Mice were euthanized by CO_2_ asphyxiation followed by cervical dislocation. The head was then removed, the skin covering the skull removed (making sure to carefully cut around the tissue surrounding the orbit), and the skull was cut down the medial line and removed along with the brain up to the cerebellum, pons, and medulla. The head was then placed in 2% paraformaldehyde overnight. The following day, the trigeminal ganglion was located [31], severed at the ophthalmic branch, and DiI crystals (1, 1—dioctadecyl-3,3,3’,3’-tetramethylindocarbocyanine perchlorate, ThermoFisher, Waltham, MA) were crushed into the ganglia using surgical tweezers (**Fig 2**). The region around the ganglia was dried using chem-wipes prior to DiI application, as DiI is a hydrophobic substance [32, 33]. The skull was then filled with 5% low-melting temperature agarose using a pipette, allowed to harden at 4⁰C for 2 minutes. The tissue was then placed in 2% paraformaldehyde and allowed to sit at 4⁰C for 26 weeks. Following this period, the eyes were enucleated, corneas isolated, stained with DAPI, and flat-mounted for imaging. Control mice, where DiI was excluded from the tissue preparation, were included in the study.

#### Imaging of DiI labeled corneal nerves and basal epithelial cells

Corneas were imaged using a DeltaVision wide field deconvolution fluorescence microscope (GE Life Sciences, Pittsburg, PA) with a 60x immersion oil lens. Corneas were then scanned for fusion events defined as a basal epithelial cell/cells with DiI labeled plasma membrane immediately adjacent to a DiI labeled stromal nerve. The central cornea was defined as the centermost 2 mm of the cornea. The remaining 1.5 mm region, defined at its edge by the limbal vasculature, was considered the peripheral cornea.

### Statistics

GraphPad Prism (GraphPad Software. San Diego, CA, USA) was used for statistical analysis and data represented as the mean ± standard error of the mean. A two-tailed Student’s t-test was performed to compare surface-to-volume ratios between penetrating and fusing nerves, while a Mann Whitney U Test (Wilcoxon Rank Sum Test) was used to compare the basal lamina pore size between the two groups. A p-value of ≤ 0.05 was considered to be statistically significant.

## Results

### SBF-SEM imaging of mouse corneal nerves revealed conventional nerve penetration as well as novel neuronal-epithelial cell fusion events

**Fig 3:**
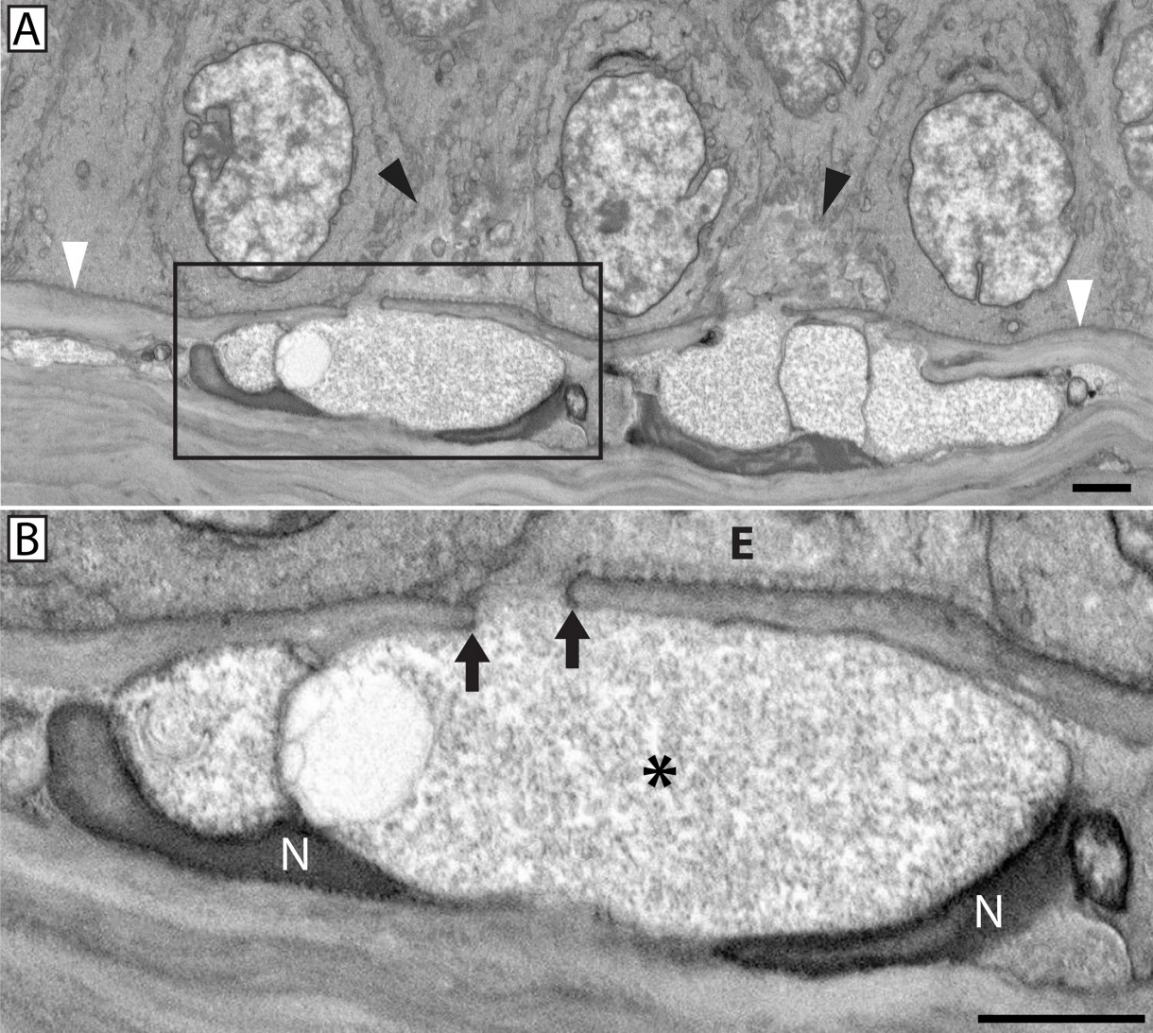
Stromal nerve fusion with the basal epithelium. A stromal nerve fused with two basal epithelial cells (black arrowheads) through two distinct pores in the basal lamina (Electron density; white arrowheads) (**A**). Enlargement of panel (**A**) inset showing magnified view of one basal lamina pore (**B**). Note the continuity between neuronal and epithelial plasma membranes at the site of fusion (arrows). Electron dense Schwann cell nuclei (N) were visible near the fusion site. The axoplasm (*) was electron translucent, lacked mitochondria and mixing between axoplasm and epithelial cell cytoplasm (E) was not evident. Scale bars = 2 μm.

**Fig 4:**
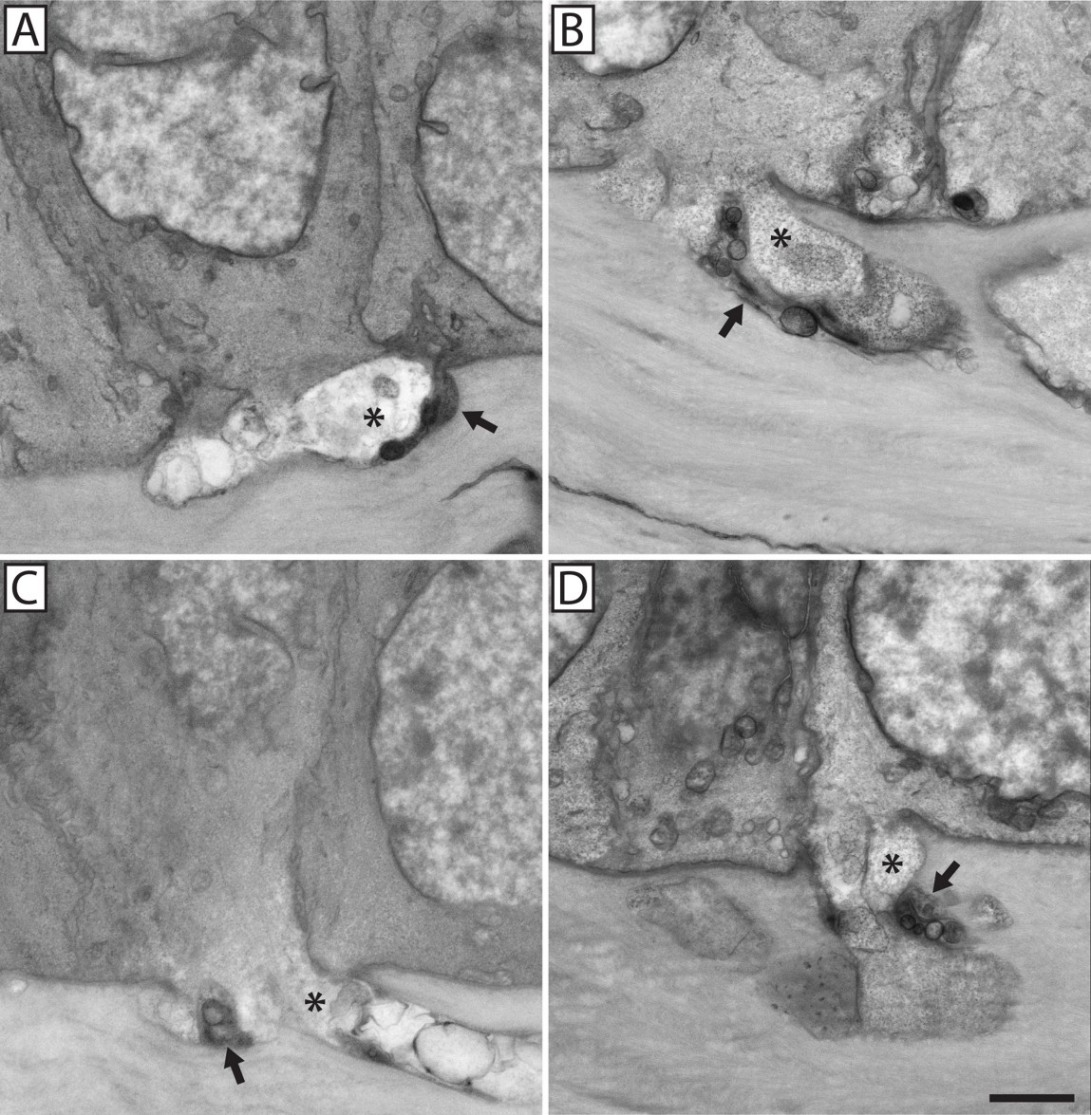
Neuronal-epithelial cell fusion involved mixed bundles of fusing and penetrating axons. Four examples of neuronal-epithelial cell fusion (**A-D**). The electron translucent portion of each nerve bundle (*) was fused with a basal epithelial cell. Penetrating nerves that continued into the epithelium (visible in Panel **A**) contributed to the sub-basal plexus and were recognized by their greater electron density (arrow). Scale bar = 2 μm.

Using SBF-SEM we were able to image conventional nerve penetration through the epithelial basal lamina, where a stromal nerve bundle containing multiple axons passes through the epithelial basal lamina to form a leash point whereby the nerve bundle gives rise to multiple smaller axonal projections which extend between epithelial cells and give rise to the sub-basal and epithelial plexuses. In addition to conventional nerve penetration through the basal lamina, a novel neuronal-epithelial cell fusion event was observed (**Fig 3A**). Nerve bundles involved in fusion contain axons whose plasma membrane is fused and continuous with that of a basal epithelial cell such that the axoplasm comes into direct contact with the cytoplasm of the fused epithelial cell (**Fig 3B**). In all cases of neuronal-epithelial cell fusion (21 total events across 10 animals), the fusing axons were accompanied by conventional penetrating axons within the same nerve bundle. In other words, these nerve bundles contained a mixture of fusing and penetrating axons. Penetrating axons were easily distinguishable amongst fusing axons as their axoplasm was characteristically electron dense compared to the diffuse, electron translucent axoplasm associated with fusing axons (**Fig 4**). Most often, a single nerve bundle fused with multiple basal epithelial cells; however, fusion with single basal epithelial cells was also observed. Whether fusion was initiated by the nerve or the epithelium could not be determined.

After the initial discovery of neuronal-epithelial cell fusion, we sought to determine the frequency and distribution of neuronal-epithelial cell fusion events using SBF-SEM on C57BL/6J mice (n = 6). Serial transverse images were collected from the central and peripheral cornea and nerves that approached the epithelial basal lamina were identified. Of 21 stromal nerve bundles that interacted with the central corneal epithelium, 9 contained axons that fused with basal epithelial cells (42.8% of nerves observed) while the remaining 12 nerve bundles only gave rise to conventional nerve penetration and leash formation. In contrast, stromal nerve bundles that engaged the basal epithelium in the peripheral cornea (21 interactions) showed no evidence of fusion as they penetrated the basal lamina and gave rise to the sub-basal and epithelial nerve plexuses.

### 3D Reconstruction of conventional nerve penetration and neuronal-epithelial cell fusion

To better characterize the ultrastructural organization of neuronal-epithelial cell fusion and document how it differed from conventional nerve penetration, SBF-SEM was used to collect serial image stacks suitable for segmentation and 3D reconstruction. When segmenting neuronal-epithelial cell fusion, care was taken to trace the electron translucent portion of the fusing axon separately from the penetrating axons with their characteristic electron dense axoplasm.

**Fig 5:**
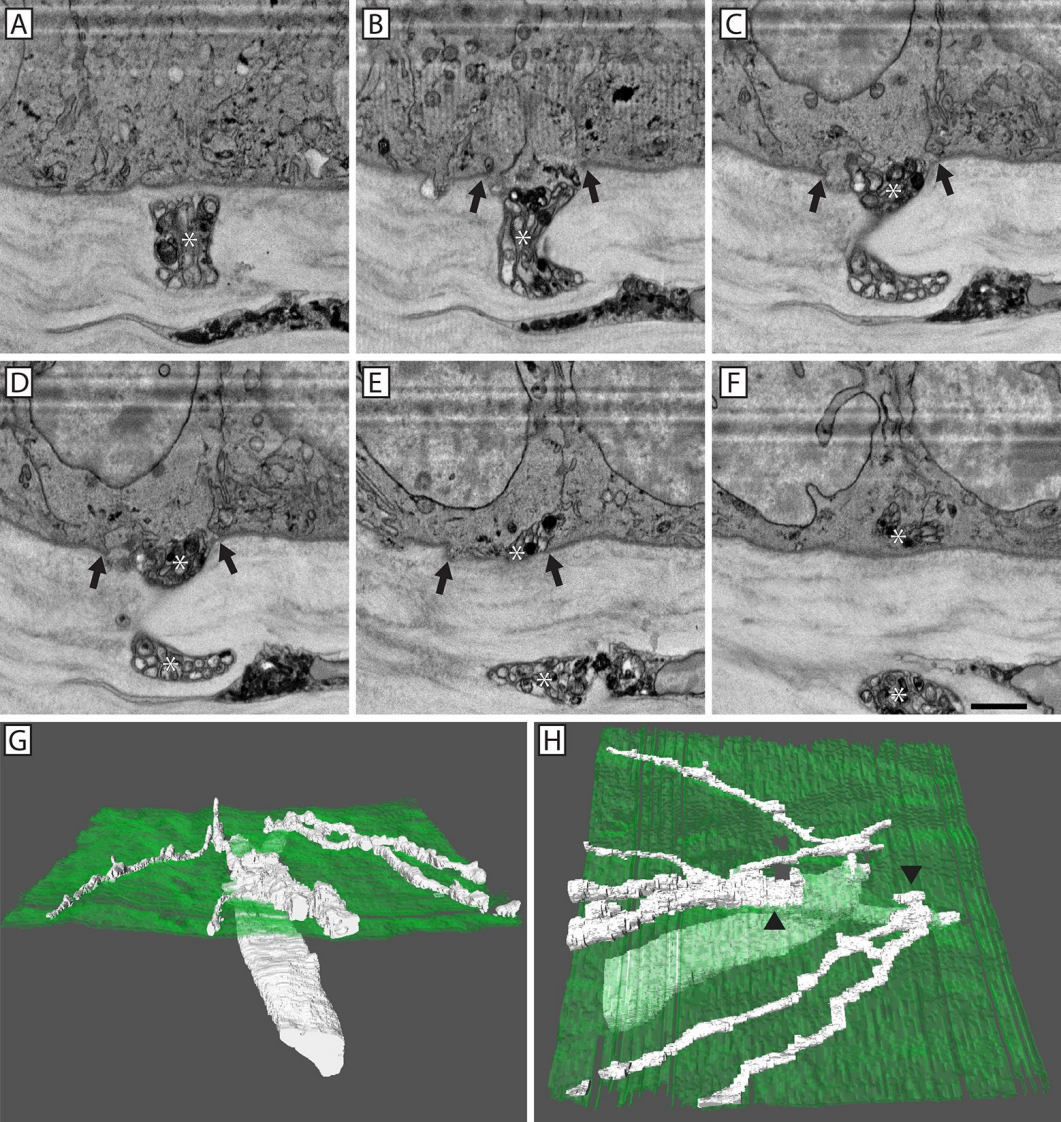
3D reconstruction of nerve penetration through the epithelial basal lamina. A series of SBF-SEM images showing a penetrating electron dense corneal nerve (*; **A-F**) that entered the epithelium through a discontinuity in the basal lamina (Panels **B-E**; arrows). A continuous basal lamina was present on either side of the penetration point (**A & F**). 3D reconstruction of the penetrating nerve (white) as it passed through the basal lamina (green; **G & H**). The nerve bifurcated prior to penetration (**H**; arrowheads). After penetrating into the corneal epithelium, both nerve branches underwent ramification. Scale bar = 2 μm.

**Fig 6:**
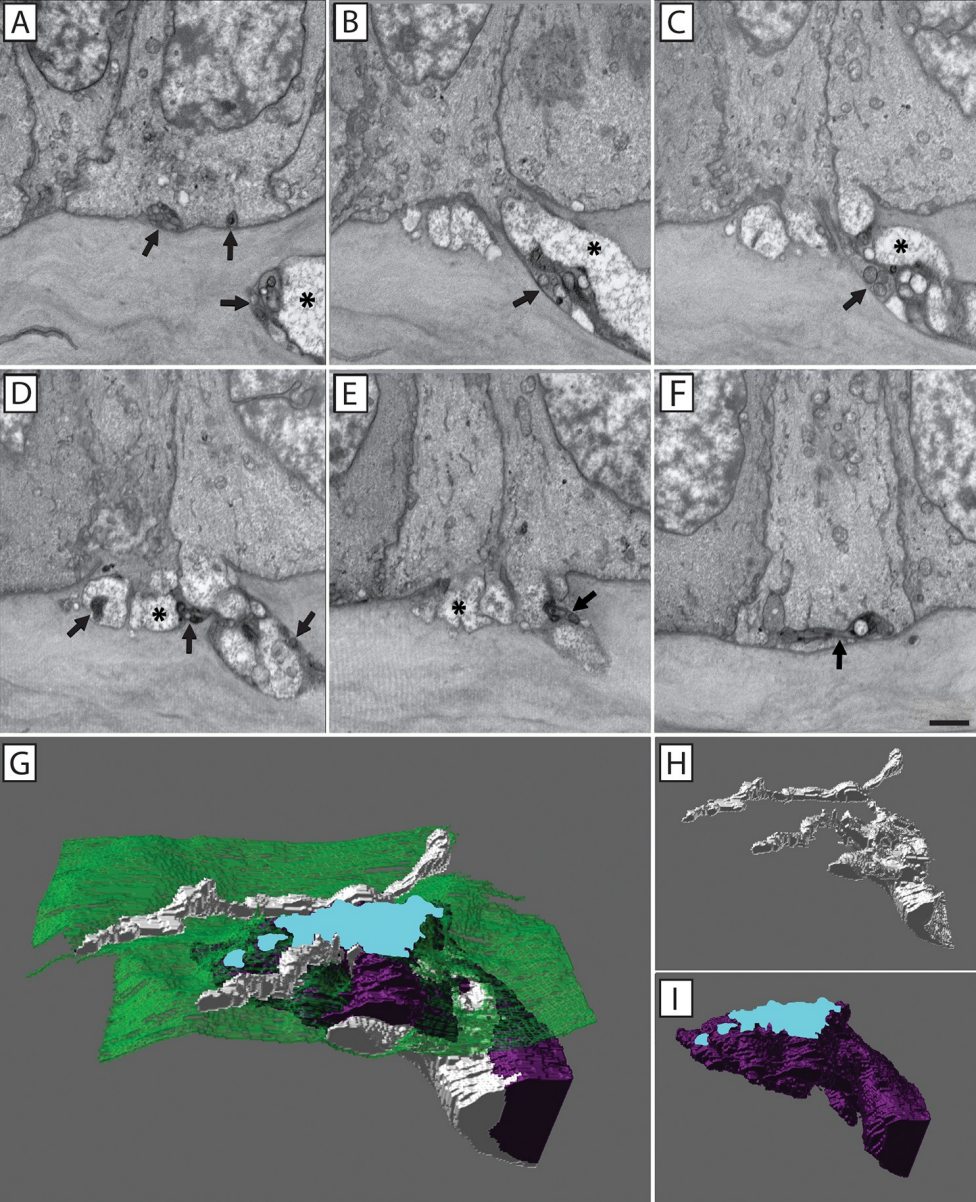
3D reconstruction of neuronal-epithelial cell fusion at the epithelial basal lamina. A series of SBF-SEM images showing a mixed nerve bundle in which fusing (*) and penetrating axons (arrows) were evident (**A-F**). The electron dense penetrating axons passed through the basal lamina and contributed to the sub-basal plexus (**F**). 3D reconstruction of a mixed nerve bundle showing fusing (purple) and penetrating (white) axons (**G**). A large fusion area (blue) denotes neuronal fusion involving three separate epithelial cells and both penetrating (**H**) and fusing (**I**) axons. Scale bar = 2 μm.

In regards to conventional penetration events, 3D reconstruction revealed a stromal nerve bundle bifurcating before extending into the epithelium through two holes, or pores, in the basal lamina (**Fig 5**). The basal epithelial cells protrude into the stroma through the basal lamina pore (**Fig 5B and 5C**) while stromal axons pass through the pore into the corneal epithelium before ramifying and establishing the sub-basal nerve plexus (**Fig 5G and 5H)**. By comparison, 3D reconstruction of a fusing nerve bundle reveals a mixed population of fusing and penetrating axons (**Fig 6**). In this example, the neuronal-epithelial cell fusion event occurred at the junction between three basal epithelial cells, commonly referred to as a Y-junction or tricellular corner. The electron dense axons within this nerve bundle passed into the basal epithelium through a pore in the basal lamina at this tricellular junction, and produced four ramifications (**Fig 6H**). The electron translucent axons within this nerve bundle did not penetrate into the epithelium, but rather fused with three separate basal epithelial cells through this basal lamina pore (**Fig 6I**).

### Nerve bundles containing neuronal-epithelial cell fusion were morphologically distinct from conventionally penetrating nerve bundles

**Fig 7:**
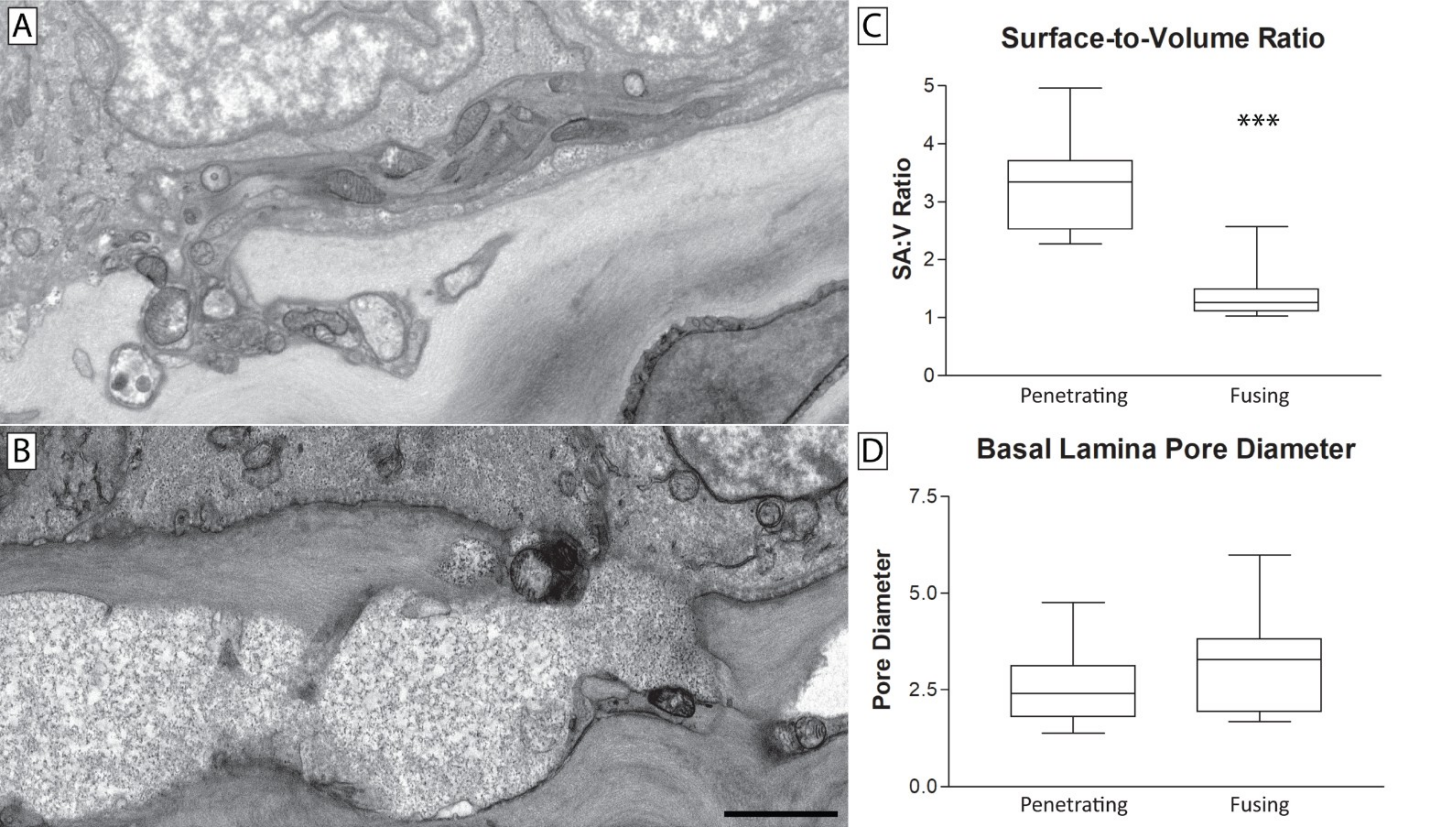
Fusing nerves had smaller surface-to-volume ratios than penetrating nerves. A penetrating nerve bundle which has passed through the epithelial basal lamina giving rise to the sub-basal plexus (**A**). The axoplasm was electron dense and contained numerous mitochondria. A nerve bundle containing fusion that has merged with a basal epithelial cell (**B**). The axoplasm was electron translucent and devoid of mitochondria. The surface-to-volume ratio of nerve bundles containing fusion were significantly smaller than that of penetrating nerve bundles consistent with their “swollen” appearance (**C**). The diameter of the basal lamina pores through which these nerves interact with the corneal epithelium was similar (**D**). Scale bars = 2 μm.

Stromal nerve bundles that interacted with the epithelium were comprised of penetrating nerves only or a mixture of penetrating and fusing nerves. In addition to their more electron dense axoplasm, the diameter of penetrating nerve bundles was noticeably smaller than their fusing counterparts (**Fig 7A and 7B**). This resulted in a marked difference in their surface-to-volume ratio (**Fig 7C**). Stromal nerve bundles that only penetrated the basal lamina and extended into the epithelium exhibited a small diameter and a high surface-to-volume ratio that was more than twice that of nerve bundles containing fusion. The smaller surface-to-volume ratio of the fused nerve bundles was consistent with the “swollen” appearance of their axoplasm (**Fig 7B**). Despite the marked differences in surface-to-volume ratios, the basal lamina pore size through which penetrating or fusing nerve bundles passed through was not different (**Fig 7D**).

Volumetric and surface data was extracted from the 3D reconstruction of fusion and conventional penetration seen in **Figs 5 and 6**. Over the same length of reconstructed nerve, the volume of axons penetrating into the epithelium was comparable, with a volume of 28.58 μm^3^ in the conventional penetration event and 24.64 μm^3^ in the fusing nerve bundle. However, the volume of fusing axons within the fusing nerve bundle accounted for three-fourths of the total nerve volume, with a volume of 75.42 μm^3^.

### Axons fused to basal epithelial cells lacked microtubules and mitochondria proximal to sites of fusion

**Fig 8:**
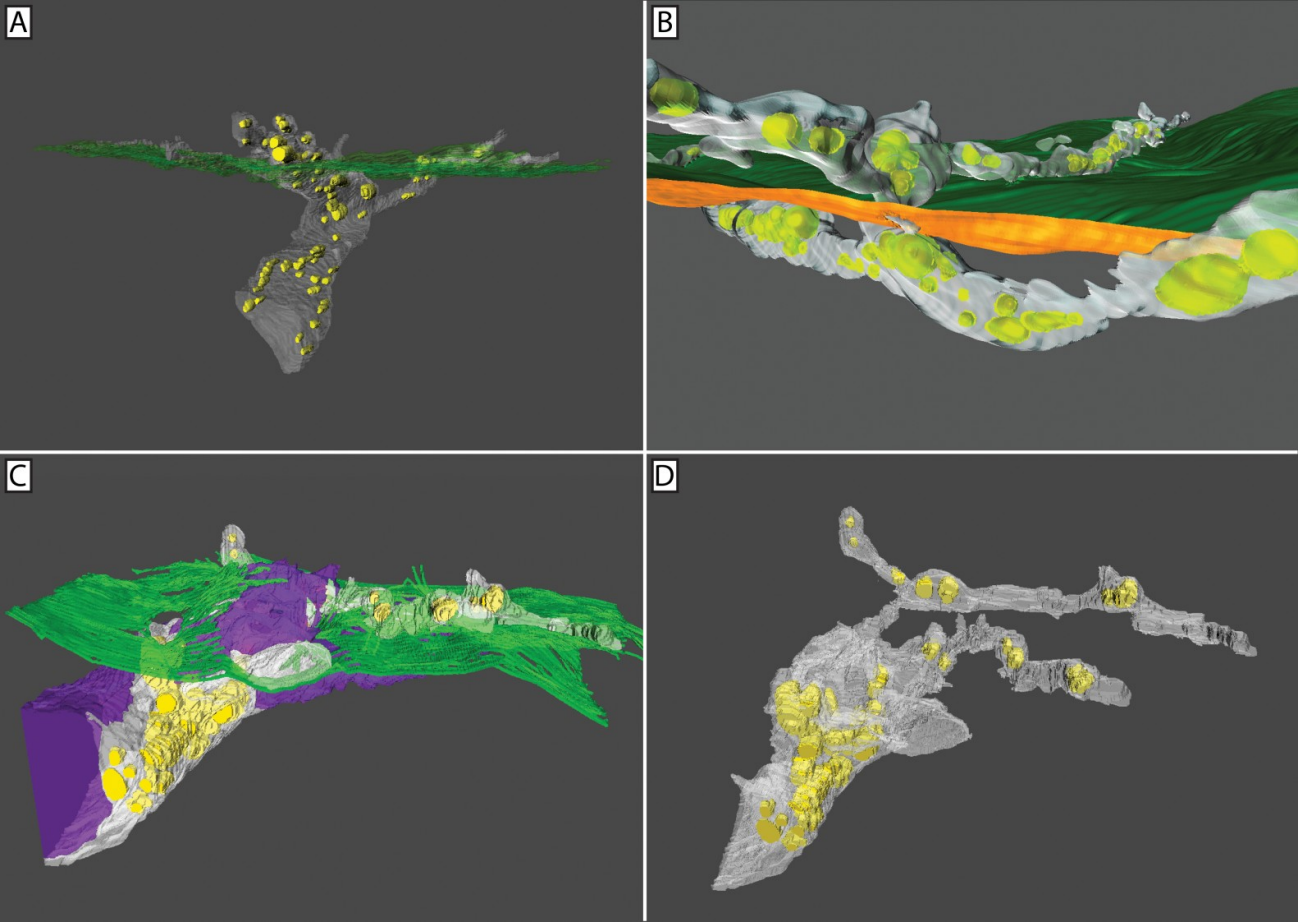
3D reconstruction confirmed fusing axons lack mitochondria at the site of fusion. Segmentation and 3D reconstruction of penetrating and fusing nerves (**A-D**). Mitochondria (yellow), penetrating axons (white), fusing axons (purple), and basal lamina (green) are shown. Conventional nerve penetration of the basal lamina involving multiple axons (**A**) or a single axon (**B**). In both cases, mitochondria were present throughout the nerve bundle on either side of the basal lamina. Mixed nerve bundle at the basal lamina showing penetrating and fusing axons (**C**). Mitochondria are clearly absent from the fusing axons. Isolation of the penetrating axons shows mitochondria to be distributed throughout the axoplasm (**D**).

**Fig 9:**
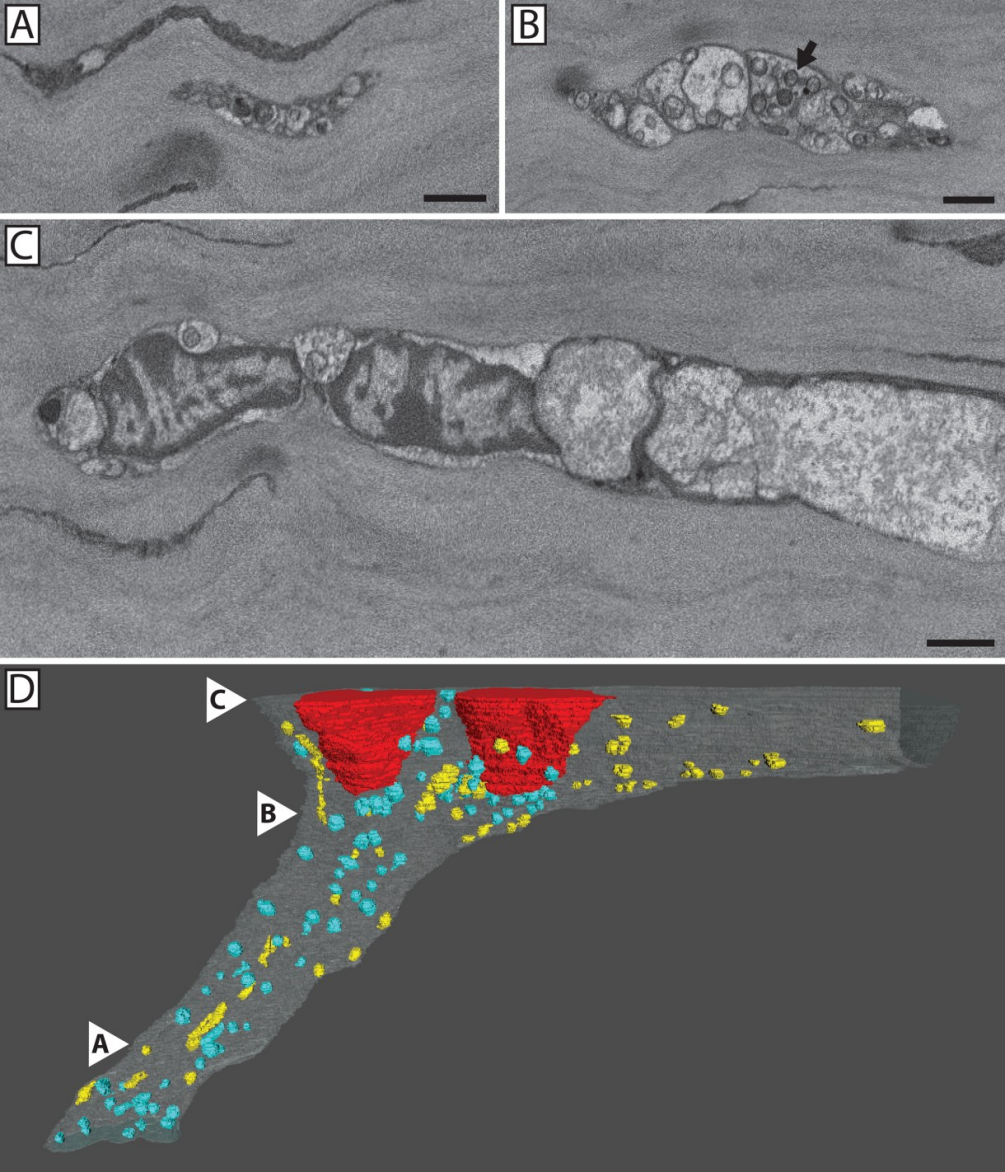
3D reconstruction showed mitochondria are present within the distal portion of fusing axons. Serial images show three levels (**A-C**) within the 3D reconstruction (**D**) of the distal portion of the mixed nerve bundle shown in **Fig 4**. The most distal portion of the nerve within the image series (**A**) was located ~60 μm distal to the site of fusion and it contained numerous mitochondria and an electron dense axoplasm. As the nerve bundle approached the fusion site, it increased in diameter (**B & C**). At ~35 um distance from the fusion site, mitochondria (blue) were no longer present in the fusing axons whereas mitochondria (yellow) were retained within the penetrating axons (**D**). White arrowheads denote the locations of panels **A-C** within the reconstructed nerve.

**Fig 10:**
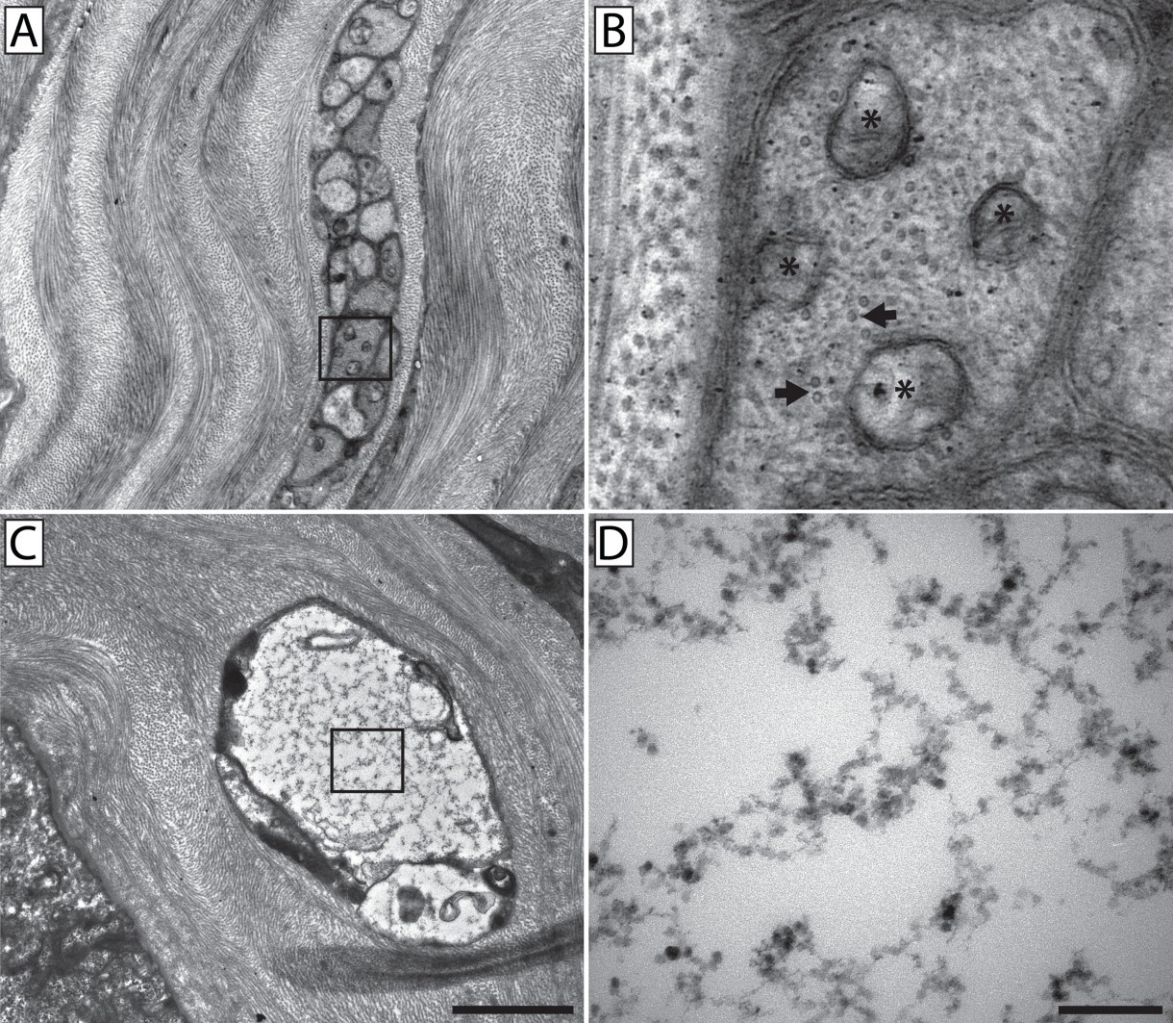
High resolution TEM showed an absence of microtubules in fusing neurons. A conventional stromal nerve bundle (**A**) in which the inset is enlarged (**B**) to show cross-sectional views of microtubules identified by their size and distinctive hollow-ring appearance (arrows). Mitochondria are also present and identified by their double-membranes and internal cristae (*). A fusing nerve bundle (**C**) with an electron translucent axoplasm in which the inset is enlarged (**D**) to show the distinct lack of microtubules and mitochondria. Scale bar for panels **A & C** = 2 μm. Scale bar for panels **B & D** = 0.2 μm.

In penetrating axons, mitochondria were distributed throughout the axoplasm of the stromal nerve as well as the ramified epithelial projections (**Fig 8A and 8B**). This was true whether the nerve bundle consisted of only penetrating axons or whether the penetrating axons were grouped alongside fusing axons, (i.e., a mixed nerve bundle). Fusing nerves contained mitochondria but only in locations distal to the fusion site (**Fig 9**). The axoplasm in close proximity to the fusion site was always devoid of mitochondria (**Fig 8C**). At higher resolution, the transmission electron microscope revealed the axoplasm of penetrating nerves was not only rich in mitochondria, but also microtubules (**Fig 10A and 10B**). By comparison, the axoplasm of fused nerves lacked microtubules near the site of fusion; the axoplasm appeared to be composed solely of dispersed and unidentifiable material (**Fig 10C and 10D**).

### Anterograde labeling confirms corneal nerve fusion with the basal epithelium

SBF-SEM imaging had proved useful for documenting nerve fusion at an ultrastructural morphologic level. Because of the novelty of the observation, we sought to confirm it using a functional method. The ultrastructure suggests the plasma membrane of the nerve fuses with the plasma membrane of the basal epithelial cell (**Fig 3**) and predicts that a lipid membrane dye, DiI, applied to the nerve should be able to diffuse into the lipid membrane of the fused epithelial cell. DiI is a commonly used neuronal tracer because it diffuses along the plasma membrane [34, 35] and cannot pass from the neuron to another cell in fixed tissue unless their plasma membranes are contiguous and this only occurs at sites of cell-cell fusion [36].

**Fig 11:**
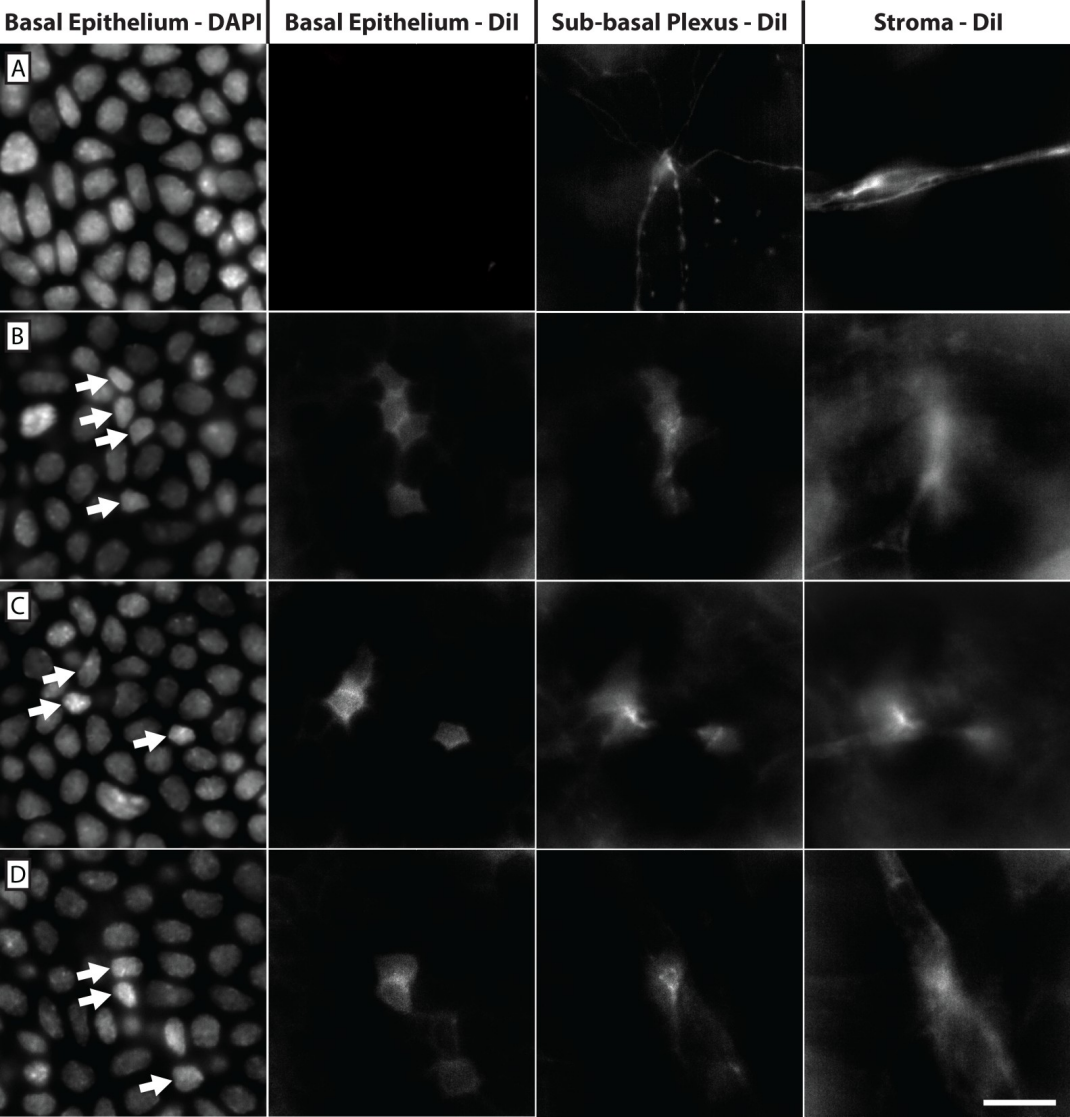
DiI applied to the trigeminal ganglion labeled corneal axons and a sub-population of basal epithelial cells. While conventional nerve penetration (**A**) showed DiI labeling extending from the stromal nerve to the sub-basal plexus, the overlying basal epithelium remained unlabeled. In addition, DiI-labeled stromal nerves approached the epithelium and labeled a sub-population of basal epithelial cells (**B-D**). DAPI staining confirmed the basal location of these epithelial cells and the arrows (column 1) denote nuclei belonging to DiI labeled basal epithelial cells (column 2). Scale bar = 2 μm.

**Fig 12:**
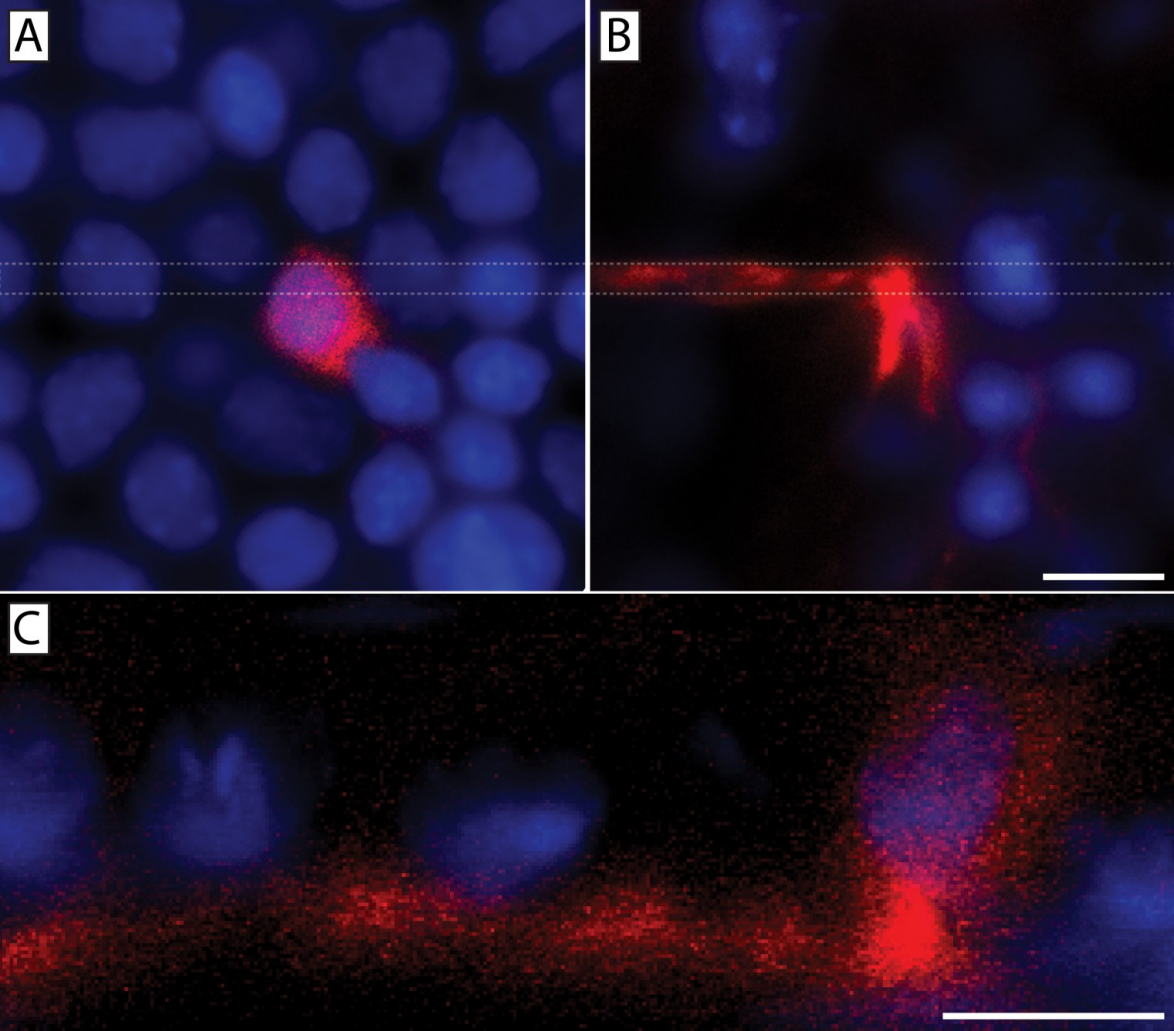
Orthogonal projection confirmed DiI transfer from corneal neuron to a single basal epithelial cell. Two fluorescence images from a Z-stack showing a DiI (red) labeled basal epithelial cell (**A**) located above a DiI labeled stromal nerve (**B**). An orthogonal slice through the stack taken between the two dashed lines is shown in panel (**C**) where the DiI labeling extended uninterrupted from the neuronal plasma membrane into the epithelial cell membrane. DAPI (blue) staining denotes cell nuclei. Scale bars = 10 μm.

We placed the lipophilic dye DiI at the trigeminal ganglia of 6 C57BL/6J mice and allowed it to diffuse along and label neuronal projections that reached into the cornea. (**Fig 11**). DiI labeling revealed axons penetrating the corneal basal lamina, ramifying, and giving rise to the sub-basal plexus (**Fig 11A**). Importantly, DiI labeling was also seen in the plasma membrane of a sub-population of basal epithelial cells associated with stromal nerves at the level of the basal lamina (**Fig 11B–11D**). DiI labeled basal epithelial cells were found primarily in the central 2 mm of each cornea. Single labeled cells as well as clusters of labeled cells were observed (**Fig 12**). Cross-sectional projections of DiI labeled epithelial cells revealed the continuity of DiI labeling from stromal nerve to basal epithelial cell (**Fig 12D**).

## Discussion

The purpose of this study was to describe and compare two types of neuronal-epithelial interactions, conventional neuronal penetration into the corneal epithelium and the novel neuronal-epithelial cell fusion that also occurs between corneal neurons and basal epithelial cells. To our knowledge, this is the first study to document fusion between neurons and basal epithelial cells in the cornea. Segmentation and reconstruction of serial images collected using SBF-SEM allowed us to unequivocally identify neuronal-epithelial cell fusion events as the merging of neuronal and epithelial plasma membranes and respective cytoplasms. Plasma membrane fusion was independently confirmed by fluorescence microscopy imaging of lipid membrane dye transfer from the neuronal plasma membrane to the epithelial cell plasma membrane. Documenting neuronal-epithelial cell fusion in the mouse cornea adds a new layer of complexity to our understanding of corneal innervation and offers new insight into the regulation of corneal nerve patterning.

Using SBF-SEM we were able to visualize the penetration of stromal nerves through the epithelial basal lamina to contribute to the epithelial plexus. These nerves were electron dense, had a high surface-to-volume ratio, and contained abundant microtubules as well as mitochondria. The high surface-to-volume ratio of these penetrating nerves is characteristic of nerves throughout the body, and conducive to the cellular processes required for neuronal signaling [37–39]. Often, nerve bundles approaching the epithelium consist of a mixed bundle of penetrating and fusing neurons. Despite the intimate contact between penetrating and fusing axons within these bundles, no obvious morphological changes were seen in the penetrating axons. Whatever the mechanism responsible for neuronal-epithelial cell fusion, it is selective even within the same nerve bundle. Penetrating axons within a bundle containing fusion appear morphologically indistinguishable from axons present within penetrating bundles. Despite the termination of fusing axons, these fusing bundles are still able to contribute to the epithelial plexus through their subpopulation of penetrating axons.

Within the cornea, neuronal-epithelial cell fusion is fairly common and occurs primarily in the central cornea between stromal nerves that are morphologically distinct from nerves that simply penetrate into the epithelium. Fusing nerves were shown to have a significantly lower surface-to-volume ratio, an electron translucent appearance, and a distinct lack of microtubules and mitochondria in close proximity to sites of fusion. Fusion was defined as the continuity between neuronal and epithelial plasma membrane such that the epithelial cytoplasm and neuronal axoplasm are in direct contact.

While this study is the first to our knowledge to describe heterotypic neuronal-epithelial fusion in normal adult tissue, the history of cell-cell fusion can be traced back to Schwann in 1839. Ironically, given that Schwann contributed so much to the study of neurons and their associated cells, he noted this cell-cell fusion between myoblasts while studying superficial dorsal muscle in pig embryos [40]. Cell-cell fusion has since been described in many other cellular systems [41–47]. A search of the literature reveals that neuron fusion has been reported to occur between neurons in the central nervous system [44], as well as between nerves and mesenchymal stem cells during development [48]. Neurons can fuse with themselves or neighboring neurons after injury, while stem cells can fuse with neurons in what is thought to be a method of cell reprogramming [45]. Giant cell formation among fusing macrophages is central to granuloma formation [49, 50] and fusion also plays a central role in sperm-egg dynamics during sexual reproduction [51, 52]. The importance of cell-cell fusion in development and disease cannot be overstated, being involved in a wide array of biological processes, ranging from fertilization to the development of bone, muscle, and placenta, it has been implicated in the immune response, tumorigenesis, as well as aspects of stem cell-mediated tissue regeneration [53–62]. Regarding heterotypical cell-cell fusion, the fusion between neurons and stem cells during development is particularly noteworthy in relation to the fusion events outlined in this paper. Within the cornea there is a population of cells known as transient amplifying cells (TACs) which retain stem-like properties. TACs retain the ability to divide as they migrate towards the cornea center [63]. While it is not known whether these fused epithelial cells are in fact TACs, this is a possibility that warrants further study.

Neuronal-epithelial cell fusion occurs within nearly half of all nerve bundles penetrating the epithelial basal lamina in the central cornea. To our knowledge, no prior electron microscopic study has identified neuronal-epithelial cell fusion in either the cornea or other tissues within the body. Two factors likely account for this, and the first is the sparse and random nature of sampling inherent in transmission electron microscopy. To this point, in 2005 it was estimated that if all material that had *ever* come into focus in all of the transmission electron microscopes worldwide were gathered together the total tissue volume would account for less than one cubic centimeter of volume [29]. The likelihood of a section passing through a corneal nerve bundle just as it penetrates or fuses with the epithelium is rare given the small size of the nerve, the small size of the tissue block and the random nature of sampling. The second factor is the 2D nature of routine transmission electron microscopy and the uncertainty of identifying a cell profile as a neuron rather than an epithelial cell, a leukocyte or a keratocyte. The interpretation of a single electron micrograph is often subjective and always open to criticism. Such is not the case with SBF-SEM where the three dimensional context allows, for the first time, accurate and unambiguous ultrastructural detection of the neuron and its interaction with the basal epithelium.

Regarding light microscopy, the lack of a body of literature on neuronal-epithelial cell fusion can be linked to two primary factors. First, without an ultrastructural understanding of the morphology of fusing nerves, any detection of neuronal-epithelial cell fusion at the light microscopic level would be difficult to interpret as such. For example, Al-Aqaba et al. may have observed neuronal fusion when noting “the termination of sub-basal nerves into characteristic bright bulb-like thickenings” roughly the size of basal epithelial cells using confocal microscopy in human corneas [64]. These characteristic bulb-like thickenings are visible, but not discussed, in several other published confocal images [65–68]. Second, the common fluorescent markers used to locate and study corneal nerves typically do not target membranes (e.g. Thy1-YFP and anti-beta-tubulin III antibody). Towards this point, detection of neuronal-epithelial cell fusion using fluorescence microscopy necessitates using a continuous plasma-membrane bound dye or antibody specific to the neuronal lipid bilayer within the corneal tissue. And while DiI administered at the trigeminal ganglion fulfills this requirement, the technical and temporal requirements for this methodology are a limiting factor in its use. For most studies of corneal nerves an endogenous fluorescent marker such as Thy1-YFP, or an easily applied fluorescent antibody such as beta-tubulin III suffice for nerve localization, are well established methodologies within the tissue, and require marginal time and effort to use [69, 70]. For this reason, anterograde DiI labeling of corneal nerves remains an esoteric technique. However, given the extensive use of DiI in the literature for studying cell-cell fusion, this methodology was uniquely suited for our purposes [71–76].

When viewed using electron microscopy, fusing nerve bundles are morphologically distinct from nerve bundles simply penetrating into the basal epithelium. Fusing neurons exhibit an electron translucent “salt and pepper” axoplasm that is devoid of mitochondria and microtubules in the cytoplasm immediately surrounding the site of fusion. Stromal nerves involved in fusion have a significantly smaller surface-to-volume ratio, indicative of a large or swollen axon. Distal to the site of fusion however, these nerve bundles are morphologically indistinguishable from other stromal nerves, containing both mitochondria and microtubules. These observations may be linked to a calcium effect. It is well known that membrane fusion is often accompanied by an increase in intracellular calcium near the site of fusion [77–80]. Increased levels of intracellular calcium have been shown to lead to the breakdown of microtubules and the inability of mitochondria to associate with kinesin and dynein (motor proteins responsible for intracellular transport), which may explain why neither mitochondria nor microtubules are present proximal to sites of neuronal-epithelial cell fusion, but can be seen in abundance distal to sites of fusion [81–83]. Without mitochondrial support or functional microtubules to traffic mitochondria and intracellular proteins near the site of fusion, axonal swelling occurs. However, the fate of these fusing axons is not known [84]. Given that fusing nerves appear morphologically typical distal to sites of fusion, the fate of these neurons cannot be assumed. In fact, similar axonal responses have been seen to be both transient and reversible in a variety of models [85–89]. It is possible that fusion with basal epithelial cells denies a subpopulation of stromal nerves the ability to innervate the epithelium, causing them to undergo a form of Wallerian degeneration followed by continued growth, and subsequent attempts to penetrate into the basal epithelium [90].

While the lack of mitochondria and microtubules near sites of fusion suggest the fused axons may be neurologically inactive, it is important to consider this alternative. If fused axons are neurologically active, gap junction communication between a fused basal epithelial cell and its neighbors would surely “short-circuit” transmission unless the gap junctions switched to a “closed” state. The switch from an “open” to a “closed” state can occur in response to a variety of stimuli, including changes in the levels of intracellular calcium, pH, transjunctional applied voltage, phosphorylation, and in response to activation of membrane receptors [91–93]. Gap junction closure would also serve to mitigate the risk of infectious agent and/or toxin transfer from basal epithelial cells into fused stromal axons. If fused axons are capable of creating action potentials, then the fused epithelial cell may function as its terminal.

While the function of neuronal-epithelial cell fusion in the cornea is open to speculation, we favor the idea that this interaction plays a role in limiting and shaping the neuronal network. The rationale behind this idea comes from noting that although the stromal nerve plexus does not change with age, the basal and epithelial nerve plexuses are constantly in flux, changing tortuosity and losing density as we age [94–99]. This suggests that axonal rearrangement occurs even in the normal, uninjured cornea. Given the relatively high frequency of fusion in the normal mouse cornea, it seems reasonable to suppose that neuronal-epithelial cell fusion is a determinant of axonal patterning which in turn would affect corneal sensitivity and epithelial proliferation (e.g., through neuropeptide release). Additionally, as the corneal epithelial cells migrate towards the central cornea, the subbasal and epithelial plexuses are dragged along with them [65]. This creates the possibility of overabundant or improper innervation of the central cornea and the necessity of neuronal rearrangement. It is possible that neuronal-epithelial cell fusion plays a role in this, and this may account for the localization of fusion events within the central cornea. Rather than the complete degeneration and loss of a neuron spanning the distance between trigeminal ganglion and corneal surface, neuronal-epithelial cell fusion would allow a neuron to maintain the integrity of its soma during the process of axonal rearrangement.

## Conclusion

Here we provide evidence for the novel neuronal-epithelial cell fusion event within the cornea. This event is primarily defined by the fusion between the plasma membrane of a stromal nerve with that of one or more basal epithelial cells such that axoplasm and cytoplasm are no longer separate. This event is morphologically distinct in that fusing nerves exhibit electron translucency, a lack of mitochondria and microtubules proximal to the site of fusion, and a significantly smaller surface-to-volume ratio. This cell-cell interaction may play a role in regulating neuronal patterning changes that accompany aging and tissue damage. Conceivably, within the cornea, neuronal fusion may influence corneal sensitivity and epithelial homeostasis throughout the life of an individual.

## Supporting information

S1 Movie Serial imaging and reconstruction of conventional nerve penetration. (MP4)

S2 Movie Serial imaging and reconstruction of neuronal-epithelial cell fusion. (MP4)

S1 Fig The resolution of membrane profiles using SBF-SEM. Stromal nerve bundle fusion with a basal epithelial cell (**A**). This image was taken at 9 kV in high vacuum. A spot size of 4.9 nm and pixel size of 7.3 nm were used, with a magnification of 37,000x. Enlargement of the uppermost inset of panel **A** reveals the double membrane of the nuclear envelope (white arrow), the single membrane of the endoplasmic reticulum (black arrow), as well as a section of the interdigitating double membrane present at the cell-cell border between the fused epithelial cell and its neighbor (black arrowheads) (**B**). Enlargement of the middle inset in panel **A** reveals a continuation of the double membrane of the nuclear envelope (white arrow), an additional portion of the single membrane of the endoplasmic reticulum (black arrow), as well as the double membrane of a mitochondrion (white arrowhead) with visible internal cristae (**C**). Enlargement of the bottommost inset in panel **A** reveals a lack of membrane between the two cells at the site of fusion, a finding common to all serial images of fusion events. If membranes were present, they would be visible as the double membrane of an axonal and epithelial cell border. The slight electron density visible is most likely accounted for by the organized cytoskeleton seen above the hemidesmosomes (Panel **A**, *) which appears to extend across the fusion site. Scale bars = 500 nm.
(TIF)

S1 File Surface-to-volume ratio and basal lamina pore diameter data. (XLSX)

## References

[1] Rozsa AJ, Beuerman RW. Density and organization of free nerve endings in the corneal epithelium of the rabbit. Pain. 1982;14(2):105–20. Epub 1982/10/01. 10.1016/0304-3959(82)90092-6 . 7177676

[2] Zhao J, Nagasaki T. A role of corneal nerves in epithelial homeostasis. Investigative ophthalmology & visual science. 2006;47:3956.

[3] Belmonte C GJ. 6: Corneal Nociceptors. Neurobiology of Nociceptors. 1996:146.

[4] Belmonte C, Aracil A, Acosta MC, Luna C, Gallar J. Nerves and sensations from the eye surface. The ocular surface. 2004;2(4):248–53. Epub 2007/01/12. . 17216099 10.1016/s1542-0124(12)70112-x

[5] Devor M. Sodium channels and mechanisms of neuropathic pain. The journal of pain: official journal of the American Pain Society. 2006;7(1 Suppl 1):S3–s12. Epub 2006/01/24. 10.1016/j.jpain.2005.09.006 . 16426998

[6] Matzner O, Devor M. Hyperexcitability at sites of nerve injury depends on voltage-sensitive Na+ channels. Journal of neurophysiology. 1994;72(1):349–59. Epub 1994/07/01. 10.1152/jn.1994.72.1.349 . 7965019

[7] Muller LJ, Marfurt CF, Kruse F, Tervo TM. Corneal nerves: structure, contents and function. Experimental eye research. 2003;76(5):521–42. Epub 2003/04/17. 10.1016/s0014-4835(03)00050-2 . 12697417

[8] Sridhar MS. Anatomy of cornea and ocular surface. Indian journal of ophthalmology. 2018;66(2):190–4. Epub 2018/01/31. 10.4103/ijo.IJO_646_17 29380756 PMC5819093

[9] Stepp MA, Tadvalkar G, Hakh R, Pal-Ghosh S. Corneal epithelial cells function as surrogate Schwann cells for their sensory nerves. Glia. 2017;65(6):851–63. Epub 2016/11/24. 10.1002/glia.23102 27878997 PMC5395310

[10] Schimmelpfennig B. Nerve structures in human central corneal epithelium. Graefe's archive for clinical and experimental ophthalmology = Albrecht von Graefes Archiv fur klinische und experimentelle Ophthalmologie. 1982;218(1):14–20. Epub 1982/01/01. 10.1007/bf02134093 . 7056476

[11] Woolf CJ. Dissecting out mechanisms responsible for peripheral neuropathic pain: implications for diagnosis and therapy. Life sciences. 2004;74(21):2605–10. Epub 2004/03/26. 10.1016/j.lfs.2004.01.003 . 15041442

[12] Muller LJ, Pels L, Vrensen GF. Ultrastructural organization of human corneal nerves. Investigative ophthalmology & visual science. 1996;37(4):476–88. Epub 1996/03/01. . 8595948

[13] Harris KM, Perry E, Bourne J, Feinberg M, Ostroff L, Hurlburt J. Uniform serial sectioning for transmission electron microscopy. The Journal of neuroscience: the official journal of the Society for Neuroscience. 2006;26(47):12101–3. Epub 2006/11/24. 10.1523/jneurosci.3994-06.2006 . 17122034 PMC6675417

[14] Gonzalez-Gonzalez O, Bech F, Gallar J, Merayo-Lloves J, Belmonte C. Functional Properties of Sensory Nerve Terminals of the Mouse Cornea. Investigative ophthalmology & visual science. 2017;58(1):404–15. Epub 2017/01/25. 10.1167/iovs.16-20033 . 28118665

[15] Lafontant PJ, Behzad AR, Brown E, Landry P, Hu N, Burns AR. Cardiac Myocyte Diversity and a Fibroblast Network in the Junctional Region of the Zebrafish Heart Revealed by Transmission and Serial Block-Face Scanning Electron Microscopy. PloS one. 2013;8(8):e72388 10.1371/journal.pone.0072388 PMC3751930. 24058412 PMC3751930

[16] Denk W, Horstmann H. Serial Block-Face Scanning Electron Microscopy to Reconstruct Three-Dimensional Tissue Nanostructure. PLoS Biology. 2004;2(11):e329 10.1371/journal.pbio.0020329 PMC524270. 15514700 PMC524270

[17] Nguyen HB, Thai TQ, Saitoh S, Wu B, Saitoh Y, Shimo S, et al Conductive resins improve charging and resolution of acquired images in electron microscopic volume imaging. Sci Rep. 2016;6:23721 Epub 2016/03/30. 10.1038/srep23721 27020327 PMC4810419

[18] Flegler SL, Heckman JW, Klomparens KL. Scanning and Transmission Electron Microscopy: An Introduction. Oxford New York: Oxford University Press, Inc.; 1993.

[19] Petrescu MS, Larry CL, Bowden RA, Williams GW, Gagen D, Li Z, et al Neutrophil interactions with keratocytes during corneal epithelial wound healing: a role for CD18 integrins. Investigative ophthalmology & visual science. 2007;48(11):5023–9. Epub 2007/10/27. 10.1167/iovs.07-0562 17962453 PMC2228250

[20] Gagen D, Laubinger S, Li Z, Petrescu MS, Brown ES, Smith CW, et al ICAM-1 mediates surface contact between neutrophils and keratocytes following corneal epithelial abrasion in the mouse. Experimental eye research. 2010;91(5):676–84. Epub 2010/08/18. 10.1016/j.exer.2010.08.007 20713042 PMC2962773

[21] Reith A, Mayhew TM. Stereology and Morphometry in Electron Microscopy: Problems and Solutions: Hemisphere Publishing Corporation; 1988.

[22] Anderson HR, Stitt AW, Gardiner TA, Archer DB. Estimation of the surface area and volume of the retinal capillary basement membrane using the stereologic method of vertical sections. Analytical and quantitative cytology and histology. 1994;16(4):253–60. Epub 1994/08/01. . 7945701

[23] Gibbons CH, Illigens BM, Wang N, Freeman R. Quantification of sweat gland innervation: a clinical-pathologic correlation. Neurology. 2009;72(17):1479–86. Epub 2009/04/29. 10.1212/WNL.0b013e3181a2e8b8 19398703 PMC2677479

[24] Knust J, Ochs M, Gundersen HJ, Nyengaard JR. Stereological estimates of alveolar number and size and capillary length and surface area in mice lungs. Anatomical record (Hoboken, NJ: 2007). 2009;292(1):113–22. Epub 2008/12/31. 10.1002/ar.20747 . 19115381

[25] Mahon GJ, Anderson HR, Gardiner TA, McFarlane S, Archer DB, Stitt AW. Chloroquine causes lysosomal dysfunction in neural retina and RPE: implications for retinopathy. Current eye research. 2004;28(4):277–84. Epub 2004/07/21. 10.1076/ceyr.28.4.277.27835 . 15259297

[26] Michel RP, Cruz-Orive LM. Application of the Cavalieri principle and vertical sections method to lung: estimation of volume and pleural surface area. Journal of microscopy. 1988;150(Pt 2):117–36. Epub 1988/05/01. 10.1111/j.1365-2818.1988.tb04603.x . 3411604

[27] Schmitz C, Hof PR. Design-based stereology in neuroscience. Neuroscience. 2005;130(4):813–31. Epub 2005/01/18. 10.1016/j.neuroscience.2004.08.050 . 15652981

[28] Weibel ER. Stereological methods in cell biology: where are we—where are we going? The journal of histochemistry and cytochemistry: official journal of the Histochemistry Society. 1981;29(9):1043–52. Epub 1981/09/01. 10.1177/29.9.7026667 . 7026667

[29] Howard CV, Reed MG. Unbiased Stereology. 2nd ed: Garland Science/BIOS Scientific Publishers; 2005.

[30] Schindelin J, Arganda-Carreras I, Frise E, Kaynig V, Longair M, Pietzsch T, et al Fiji: an open-source platform for biological-image analysis. Nat Methods. 2012;9(7):676–82. Epub 2012/06/30. 10.1038/nmeth.2019 22743772 PMC3855844

[31] Jackson M, Tourtellotte W. Neuron Culture from Mouse Superior Cervical Ganglion. Bio-protocol. 2014;4(2). Epub 2014/01/20. 27054145 10.21769/bioprotoc.1035 PMC4819978

[32] Hofmann MH, Bleckmann H. Effect of temperature and calcium on transneuronal diffusion of DiI in fixed brain preparations. J Neurosci Methods. 1999;88(1):27–31. Epub 1999/06/24. 10.1016/s0165-0270(99)00007-2 . 10379576

[33] Murphy MC, Fox EA. Anterograde tracing method using DiI to label vagal innervation of the embryonic and early postnatal mouse gastrointestinal tract. J Neurosci Methods. 2007;163(2):213–25. Epub 2007/04/10. 10.1016/j.jneumeth.2007.03.001 17418900 PMC1974840

[34] Balice-Gordon RJ, Chua CK, Nelson CC, Lichtman JW. Gradual loss of synaptic cartels precedes axon withdrawal at developing neuromuscular junctions. Neuron. 1993;11(5):801–15. Epub 1993/11/01. 10.1016/0896-6273(93)90110-d . 8240805

[35] Godement P, Vanselow J, Thanos S, Bonhoeffer F. A study in developing visual systems with a new method of staining neurones and their processes in fixed tissue. Development. 1987;101(4):697–713. Epub 1987/12/01. . 2460302 10.1242/dev.101.4.697

[36] Honig MG, Hume RI. Fluorescent carbocyanine dyes allow living neurons of identified origin to be studied in long-term cultures. J Cell Biol. 1986;103(1):171–87. Epub 1986/07/01. 10.1083/jcb.103.1.171 2424918 PMC2113786

[37] Doane MG. Fluorometric measurement of pyridine nucleotide reduction in the giant axon of the squid. The Journal of general physiology. 1967;50(11):2603–32. Epub 1967/12/01. 10.1085/jgp.50.11.2603 4384698 PMC2225669

[38] Yu DY, Cringle SJ, Balaratnasingam C, Morgan WH, Yu PK, Su EN. Retinal ganglion cells: Energetics, compartmentation, axonal transport, cytoskeletons and vulnerability. Prog Retin Eye Res. 2013;36:217–46. Epub 2013/07/31. 10.1016/j.preteyeres.2013.07.001 . 23891817

[39] Waxman SG, Kocsis JD, Stys PK. The Axon: Structure, Function and Pathophysiology. Oxford, New York: Oxford University Press; 1995. 691 p.

[40] Schwann TH. Microscopical Research into the Accordance in the Structure and Growth of Animals and Plants. London: The Syndenham Society; 1847. 314 p. 10.1002/j.1550-8528.1993.tb00021.x 16350590

[41] Ogle BM, Cascalho M, Platt JL. Biological implications of cell fusion. Nature reviews Molecular cell biology. 2005;6(7):567–75. Epub 2005/06/16. 10.1038/nrm1678 . 15957005

[42] Huda F, Fan Y, Suzuki M, Konno A, Matsuzaki Y, Takahashi N, et al Fusion of Human Fetal Mesenchymal Stem Cells with "Degenerating" Cerebellar Neurons in Spinocerebellar Ataxia Type 1 Model Mice. PloS one. 2016;11(11):e0164202 Epub 2016/11/02. 10.1371/journal.pone.0164202 27802273 PMC5089746

[43] Ackman JB, Siddiqi F, Walikonis RS, LoTurco JJ. Fusion of microglia with pyramidal neurons after retroviral infection. The Journal of neuroscience: the official journal of the Society for Neuroscience. 2006;26(44):11413–22. Epub 2006/11/03. 10.1523/jneurosci.3340-06.2006 . 17079670 PMC6674527

[44] Sotnikov OS. Use of Cell Culture to Prove Syncytial Connection and Fusion of Neurons. Ceccherini-Nelli DL, editor: InTech; 2012.

[45] Ambrosi DJ, Rasmussen TP. Reprogramming mediated by stem cell fusion. Journal of cellular and molecular medicine. 2005;9(2):320–30. Epub 2005/06/21. 10.1111/j.1582-4934.2005.tb00358.x . 15963252 PMC6740314

[46] Bittner GD, Sengelaub DR, Trevino RC, Peduzzi JD, Mikesh M, Ghergherehchi CL, et al The curious ability of polyethylene glycol fusion technologies to restore lost behaviors after nerve severance. Journal of neuroscience research. 2016;94(3):207–30. Epub 2015/11/04. 10.1002/jnr.23685 26525605 PMC4981502

[47] Kim JH, Jin P, Duan R, Chen EH. Mechanisms of myoblast fusion during muscle development. Current opinion in genetics & development. 2015;32:162–70. Epub 2015/05/20. 10.1016/j.gde.2015.03.006 25989064 PMC4508005

[48] Ying QL, Nichols J, Evans EP, Smith AG. Changing potency by spontaneous fusion. Nature. 2002;416(6880):545–8. Epub 2002/04/05. 10.1038/nature729 . 11932748

[49] Brodbeck WG, Anderson JM. GIANT CELL FORMATION AND FUNCTION. Current opinion in hematology. 2009;16(1):53–7. 10.1097/MOH.0b013e32831ac52e PMC2679387. 19057205 PMC2679387

[50] Vignery A. Osteoclasts and giant cells: macrophage-macrophage fusion mechanism. International journal of experimental pathology. 2000;81(5):291–304. Epub 2001/02/13. 10.1111/j.1365-2613.2000.00164.x 11168677 PMC2517739

[51] Primakoff P, Myles DG. Penetration, adhesion, and fusion in mammalian sperm-egg interaction. Science (New York, NY). 2002;296(5576):2183–5. Epub 2002/06/22. 10.1126/science.1072029 . 12077404

[52] Stein KK, Primakoff P, Myles D. Sperm-egg fusion: events at the plasma membrane. Journal of cell science. 2004;117(Pt 26):6269–74. Epub 2004/12/14. 10.1242/jcs.01598 . 15591242

[53] Chen EH, Olson EN. Unveiling the mechanisms of cell-cell fusion. Science (New York, NY). 2005;308(5720):369–73. Epub 2005/04/16. 10.1126/science.1104799 . 15831748

[54] Abmayr SM, Balagopalan L, Galletta BJ, Hong SJ. Cell and molecular biology of myoblast fusion. International review of cytology. 2003;225:33–89. Epub 2003/04/17. 10.1016/s0074-7696(05)25002-7 . 12696590

[55] Chen EH, Olson EN. Towards a molecular pathway for myoblast fusion in Drosophila. Trends in cell biology. 2004;14(8):452–60. Epub 2004/08/17. 10.1016/j.tcb.2004.07.008 . 15308212

[56] Horsley V, Pavlath GK. Forming a multinucleated cell: molecules that regulate myoblast fusion. Cells, tissues, organs. 2004;176(1–3):67–78. Epub 2004/01/28. 10.1159/000075028 . 14745236

[57] Potgens AJ, Schmitz U, Bose P, Versmold A, Kaufmann P, Frank HG. Mechanisms of syncytial fusion: a review. Placenta. 2002;23 Suppl A:S107–13. Epub 2002/04/30. 10.1053/plac.2002.0772 . 11978067

[58] Wagers AJ, Weissman IL. Plasticity of adult stem cells. Cell. 2004;116(5):639–48. Epub 2004/03/10. 10.1016/s0092-8674(04)00208-9 . 15006347

[59] Pomerantz J, Blau HM. Nuclear reprogramming: a key to stem cell function in regenerative medicine. Nature cell biology. 2004;6(9):810–6. Epub 2004/09/02. 10.1038/ncb0904-810 . 15340448

[60] Brukman NG, Uygur B, Podbilewicz B, Chernomordik LV. How cells fuse. J Cell Biol. 2019;218(5):1436–51. Epub 2019/04/03. 10.1083/jcb.201901017 . 30936162 PMC6504885

[61] Pfannkuche K. Cell Fusion: Overviews and Methods. 2nd ed Springer New York: Humana Press; 2015.

[62] Vassilopoulos G, Russell DW. Cell fusion: an alternative to stem cell plasticity and its therapeutic implications. Current opinion in genetics & development. 2003;13(5):480–5. Epub 2003/10/11. 10.1016/s0959-437x(03)00110-2 . 14550412

[63] Daniels JT, Dart JK, Tuft SJ, Khaw PT. Corneal stem cells in review. Wound repair and regeneration: official publication of the Wound Healing Society [and] the European Tissue Repair Society. 2001;9(6):483–94. Epub 2002/03/19. . 11896990 10.1046/j.1524-475x.2001.00483.x

[64] Al-Aqaba MA, Alomar T, Miri A, Fares U, Otri AM, Dua HS. Ex vivo confocal microscopy of human corneal nerves. The British journal of ophthalmology. 2010;94(9):1251–7. Epub 2010/06/30. 10.1136/bjo.2009.178244 . 20584714

[65] Patel DV, McGhee CN. In vivo laser scanning confocal microscopy confirms that the human corneal sub-basal nerve plexus is a highly dynamic structure. Investigative ophthalmology & visual science. 2008;49(8):3409–12. Epub 2008/04/29. 10.1167/iovs.08-1951 . 18441297

[66] Petroll WM, Robertson DM. In Vivo Confocal Microscopy of the Cornea: New Developments in Image Acquisition, Reconstruction, and Analysis Using the HRT-Rostock Corneal Module. The ocular surface. 2015;13(3):187–203. Epub 2015/05/23. 10.1016/j.jtos.2015.05.002 PubMed Central PMCID: PMC4499020. 25998608 PMC4499020

[67] Patel DV, McGhee CN. Mapping of the normal human corneal sub-Basal nerve plexus by in vivo laser scanning confocal microscopy. Investigative ophthalmology & visual science. 2005;46(12):4485–8. Epub 2005/11/24. 10.1167/iovs.05-0794 . 16303938

[68] Auran JD, Koester CJ, Kleiman NJ, Rapaport R, Bomann JS, Wirotsko BM, et al Scanning slit confocal microscopic observation of cell morphology and movement 10.1016/s0161-6420(95)31057-3 the normal human anterior cornea. Ophthalmology. 1995;102(1):33–41. Epub 1995/01/01. 7831039

[69] Feng G, Mellor RH, Bernstein M, Keller-Peck C, Nguyen QT, Wallace M, et al Imaging neuronal subsets in transgenic mice expressing multiple spectral variants of GFP. Neuron. 2000;28(1):41–51. Epub 2000/11/22. 10.1016/s0896-6273(00)00084-2 . 11086982

[70] Sullivan KF, Cleveland DW. Identification of conserved isotype-defining variable region sequences for four vertebrate beta tubulin polypeptide classes. Proceedings of the National Academy of Sciences of the United States of America. 1986;83(12):4327–31. 10.1073/pnas.83.12.4327 . 3459176 PMC323725

[71] Spotl L, Sarti A, Dierich MP, Most J. Cell membrane labeling with fluorescent dyes for the demonstration of cytokine-induced fusion between monocytes and tumor cells. Cytometry. 1995;21(2):160–9. Epub 1995/10/01. 10.1002/cyto.990210208 . 8582236

[72] Wojcieszyn JW, Schlegel RA, Lumley-Sapanski K, Jacobson KA. Studies on the mechanism of polyethylene glycol-mediated cell fusion using fluorescent membrane and cytoplasmic probes. J Cell Biol. 1983;96(1):151–9. Epub 1983/01/01. 10.1083/jcb.96.1.151 6826645 PMC2112265

[73] Chang DC. Cell poration and cell fusion using an oscillating electric field. Biophys J. 1989;56(4):641–52. 10.1016/S0006-3495(89)82711-0 . 2819230 PMC1280520

[74] Chi M, Xie W, Liu Y, Wen H, Zhao L, Song Y, et al Mutations in the DI-DII linker of the NDV fusion protein conferred hemagglutinin-neuraminidase-independent cell fusion promotion. The Journal of general virology. 2019;100(6):958–67. Epub 2019/05/30. 10.1099/jgv.0.001278 . 31140969

[75] Anantharam A, Axelrod D, Holz RW. Real-time imaging of plasma membrane deformations reveals pre-fusion membrane curvature changes and a role for dynamin in the regulation of fusion pore expansion. Journal of neurochemistry. 2012;122(4):661–71. Epub 2012/06/08. 10.1111/j.1471-4159.2012.07816.x 22671293 PMC3408088

[76] Hodor PG, Ettensohn CA. The dynamics and regulation of mesenchymal cell fusion in the sea urchin embryo. Developmental biology. 1998;199(1):111–24. Epub 1998/07/24. 10.1006/dbio.1998.8924 . 9676196

[77] Giordano-Santini R, Linton C, Hilliard MA. Cell-cell fusion in the nervous system: Alternative mechanisms of development, injury, and repair. Seminars in cell & developmental biology. 2016;60:146–54. Epub 2016/07/05. 10.1016/j.semcdb.2016.06.019 . 27375226

[78] Hay JC. Calcium: a fundamental regulator of intracellular membrane fusion? EMBO reports. 2007;8(3):236–40. Epub 2007/03/03. 10.1038/sj.embor.7400921 17330068 PMC1808041

[79] Alvarez-Dolado M, Pardal R, Garcia-Verdugo JM, Fike JR, Lee HO, Pfeffer K, et al Fusion of bone-marrow-derived cells with Purkinje neurons, cardiomyocytes and hepatocytes. Nature. 2003;425(6961):968–73. Epub 2003/10/14. 10.1038/nature02069 . 14555960

[80] Park JY, Jang SY, Shin YK, Koh H, Suh DJ, Shinji T, et al Mitochondrial swelling and microtubule depolymerization are associated with energy depletion in axon degeneration. Neuroscience. 2013;238:258–69. Epub 2013/03/15. 10.1016/j.neuroscience.2013.02.033 . 23485808

[81] Kramer T, Enquist LW. Alphaherpesvirus infection disrupts mitochondrial transport in neurons. Cell host & microbe. 2012;11(5):504–14. Epub 2012/05/23. 10.1016/j.chom.2012.03.005 22607803 PMC3358700

[82] Baas PW, Rao AN, Matamoros AJ, Leo L. Stability properties of neuronal microtubules. Cytoskeleton (Hoboken, NJ). 2016;73(9):442–60. Epub 2016/02/19. 10.1002/cm.21286 26887570 PMC5541393

[83] Hunter DR, Haworth RA, Southard JH. Relationship between configuration, function, and permeability in calcium-treated mitochondria. J Biol Chem. 1976;251(16):5069–77. Epub 1976/08/25. . 134035

[84] Coleman M. Axon degeneration mechanisms: commonality amid diversity. Nature reviews Neuroscience. 2005;6(11):889–98. Epub 2005/10/15. 10.1038/nrn1788 . 16224497

[85] Nikic I, Merkler D, Sorbara C, Brinkoetter M, Kreutzfeldt M, Bareyre FM, et al A reversible form of axon damage in experimental autoimmune encephalomyelitis and multiple sclerosis. Nature medicine. 2011;17(4):495–9. Epub 2011/03/29. 10.1038/nm.2324 . 21441916

[86] Caillaud M, Richard L, Vallat JM, Desmouliere A, Billet F. Peripheral nerve regeneration and intraneural revascularization. Neural regeneration research. 2019;14(1):24–33. Epub 2018/12/12. 10.4103/1673-5374.243699 30531065 PMC6263011

[87] DeFrancesco-Lisowitz A, Lindborg JA, Niemi JP, Zigmond RE. The neuroimmunology of degeneration and regeneration in the peripheral nervous system. Neuroscience. 2015;302:174–203. Epub 2014/09/23. 10.1016/j.neuroscience.2014.09.027 25242643 PMC4366367

[88] Scheib J, Hoke A. Advances in peripheral nerve regeneration. Nature reviews Neurology. 2013;9(12):668–76. Epub 2013/11/13. 10.1038/nrneurol.2013.227 . 24217518

[89] Avery MA, Rooney TM, Pandya JD, Wishart TM, Gillingwater TH, Geddes JW, et al WldS prevents axon degeneration through increased mitochondrial flux and enhanced mitochondrial Ca2+ buffering. Current biology: CB. 2012;22(7):596–600. Epub 2012/03/20. 10.1016/j.cub.2012.02.043 22425157 PMC4175988

[90] Stoll G, Jander S, Myers RR. Degeneration and regeneration of the peripheral nervous system: from Augustus Waller's observations to neuroinflammation. Journal of the peripheral nervous system: JPNS. 2002;7(1):13–27. Epub 2002/04/10. . 11939348 10.1046/j.1529-8027.2002.02002.x

[91] Oshima A. Structure and closure of connexin gap junction channels. FEBS letters. 2014;588(8):1230–7. Epub 2014/02/05. 10.1016/j.febslet.2014.01.042 . 24492007

[92] Thimm J, Mechler A, Lin H, Rhee S, Lal R. Calcium-dependent open/closed conformations and interfacial energy maps of reconstituted hemichannels. J Biol Chem. 2005;280(11):10646–54. Epub 2004/12/24. 10.1074/jbc.M412749200 . 15615707

[93] Herve JC, Derangeon M. Gap-junction-mediated cell-to-cell communication. Cell Tissue Res. 2013;352(1):21–31. Epub 2012/09/04. 10.1007/s00441-012-1485-6 . 22940728

[94] Patel DV, McGhee CN. In vivo confocal microscopy of human corneal nerves in health, in ocular and systemic disease, and following corneal surgery: a review. The British journal of ophthalmology. 2009;93(7):853–60. Epub 2008/11/21. 10.1136/bjo.2008.150615 . 19019923

[95] Cruzat A, Qazi Y, Hamrah P. In Vivo Confocal Microscopy of Corneal Nerves in Health and Disease. The ocular surface. 2017;15(1):15–47. Epub 2016/10/25. 10.1016/j.jtos.2016.09.004 27771327 PMC5512932

[96] Simsek C, Kojima T, Nagata T, Dogru M, Tsubota K. Changes in Murine Subbasal Corneal Nerves After Scopolamine-Induced Dry Eye Stress Exposure. Investigative ophthalmology & visual science. 2019;60(2):615–23. Epub 2019/02/09. 10.1167/iovs.18-26318 . 30735229

[97] Hillenaar T, van Cleynenbreugel H, Remeijer L. How normal is the transparent cornea? Effects of aging on corneal morphology. Ophthalmology. 2012;119(2):241–8. Epub 2011/11/01. 10.1016/j.ophtha.2011.07.041 . 22035579

[98] Niederer RL, Perumal D, Sherwin T, McGhee CNJ. Age-related differences in the normal human cornea: a laser scanning in vivo confocal microscopy study. The British journal of ophthalmology. 2007;91(9):1165–9. Epub 03/27. 10.1136/bjo.2006.112656 . 17389741 PMC1954900

[99] Reichard M, Weiss H, Poletti E, Ruggeri A, Guthoff RF, Stachs O, et al Age-Related Changes in Murine Corneal Nerves. Current eye research. 2016;41(8):1021–8. Epub 2015/12/09. 10.3109/02713683.2015.1088952 . 26642890

