# Supplementary figures and images for: Serial block-face scanning electron microscopy reveals neuronal-epithelial cell fusion in the mouse cornea
Source: PLoS One. 2019 Nov 13;14(11):e0224434. doi: 10.1371/journal.pone.0224434 (PMC6853292; doi:10.1371/journal.pone.0224434)

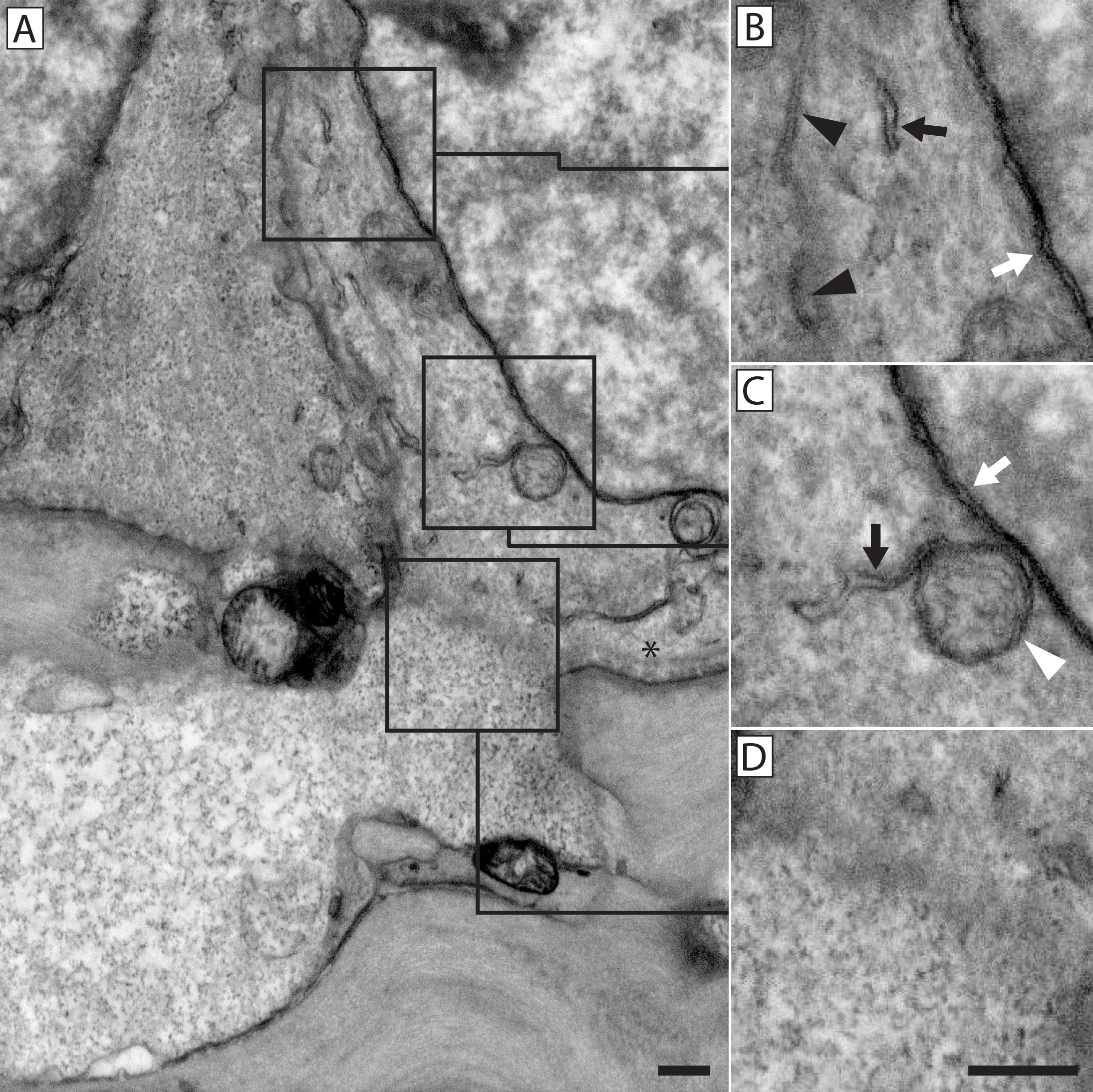

Supplement: S1 Fig — Stromal nerve bundle fusion with a basal epithelial cell (A). This image was taken at 9 kV in high vacuum. A spot size of 4.9 nm and pixel size of 7.3 nm were used, with a magnification of 37,000x. Enlargement of the uppermost inset of panel A reveals the double membrane of the nuclear envelope (white arrow), the single membrane of the endoplasmic reticulum (black arrow), as well as a section of the interdigitating double membrane present at the cell-cell border between the fused epithelial cell and its neighbor (black arrowheads) (B). Enlargement of the middle inset in panel A reveals a continuation of the double membrane of the nuclear envelope (white arrow), an additional portion of the single membrane of the endoplasmic reticulum (black arrow), as well as the double membrane of a mitochondrion (white arrowhead) with visible internal cristae (C). Enlargement of the bottommost inset in panel A reveals a lack of membrane between the two cells at the site of fusion, a finding common to all serial images of fusion events. If membranes were present, they would be visible as the double membrane of an axonal and epithelial cell border. The slight electron density visible is most likely accounted for by the organized cytoskeleton seen above the hemidesmosomes (Panel A, *) which appears to extend across the fusion site. Scale bars = 500 nm. (TIF) [file pone.0224434.s003.tif]
